# Modern genomic and omics-based technologies for millet breeding and genetic improvement

**DOI:** 10.3389/fpls.2026.1782766

**Published:** 2026-05-20

**Authors:** Anand Kumar, Pandiyan Muthuramalingam, Laxmidas Verma, Reetesh Kumar, Naveen Kumar, Jyotsna Misra, Karthikeyan Ravi, Hyunsuk Shin, Manikandan Ramesh

**Affiliations:** 1 Faculty of Agricultural Sciences, GLA University, Mathura, Uttar Pradesh, India; 2 Department of GreenBio Science, College of Agriculture and Life Sciences, Gyeongsang National University, Jinju, Republic of Korea; 3 Department of Genetics and Plant Breeding, Acharya Narendra Deva University of Agriculture and Technology, Kumarganj, Ayodhya, India; 4 Department of Biotechnology and Bioengineering, School of Biosciences & Technology, Galgotias University, Greater Noida, Gautam Buddha Nagar, India; 5 School of Basic Sciences, Galgotias University, Greater Noida, Gautam Buddha Nagar, India; 6 Department of Biotechnology, Graphic Era Deemed to be University, Dehradun, Uttarakhand, India; 7 Centre for Herbal Pharmacology and Environmental Sustainability, Chettinad Hospital and Research Institute, Chettinad Academy of Research and Education, Kelambakkam, Tamil Nadu, India; 8 Department of Biotechnology, Alagappa University, Karaikudi, Tamil Nadu, India

**Keywords:** CRISPR/Cas9, genetic engineering, global food security, machine learning, millets, omics approaches, pangenome, speed breeding

## Abstract

Millets are a diverse group of small seeded grasses that have long served as vital staple foods and forage crops across a wide range of agro-ecological regions. Known for their exceptional adaptability to marginal and resource poor environments, millets have historically supported farming communities in arid and semi-arid regions. Despite these advantages, they remain underutilized in modern agriculture due to limited genomic resources, harsh growing conditions, and insufficient technological support for their improvement. However, growing concerns over climate change, malnutrition and the need for sustainable agriculture have renewed global scientific interest in millet research and breeding. Recent breakthroughs in molecular biology such as marker-assisted selection (MAS), Genome-wide association studies (GWAS), genomic selection (GS), genetic engineering, omics technologies, speed breeding, and machine learning (ML) have significantly transformed the landscape of millet improvement. Advances in MAS, high-throughput genotyping, transcriptomics, proteomics, metabolomics, and phenomics have enabled more profound insights into the genetic architecture of key agronomic traits. These tools have facilitated the identification of genes, regulatory networks, and metabolic pathways governing drought tolerance, nutrient use efficiency, disease resistance and other essential stress responses. The integration of next-generation sequencing and comparative genomics has further expanded millet research through the development of reference genomes, pangenomes, and comprehensive germplasm characterizations. Pangenomic approaches, in particular, have uncovered structural variations and novel alleles that contribute to phenotypic diversity, offering valuable targets for breeding climate-resilient cultivars. High-resolution phenomic platforms have enhanced the precision of trait evaluation, enabling rapid screening of large populations under diverse environmental conditions. Additionally, genome editing technologies, especially CRISPR/Cas systems and multiplex CRISPR/Cas, have opened new avenues for precise genetic improvement by enabling targeted gene modification to enhance stress resilience and yield traits. Therefore, these integrated omics-driven and molecular breeding strategies are reshaping the millet improvement. With modern biotechnological innovations, researchers are now better equipped to develop high-yielding, nutrient-rich and climate-resilient millet cultivars. These advancements position millets as strategic crops that can strengthen global food and nutritional security while promoting sustainable agricultural systems in the face of mounting environmental challenges.

## 1 Introduction

The production of cereal grains has reached new heights worldwide and cereals play a significant role as the primary source of food in the human diet (Poole et al., 2022). However, the world population faces numerous challenges in feeding the human diet, including an insufficient supply, climatic variations, increasing food prices and environmental degradation (Daszkiewicz, 2022). These unfavorable conditions are challenging farmers’ ability to grow the cereals required and indicate the need to identify a suitable cereal crop to meet global food requirements (Neupane et al., 2022). In this regard, millets could be an alternative option for providing the dietary needs of a growing population. Millets are known as important cereal grains belonging to the highly diverse grass family cultivated across Asia and Africa for thousands of years as major food staples and livestock forage (Muskan et al., 2025). In India, different types of millets, including pearl millet (*Pennisetum glaucum*), sorghum (*Sorghum bicolor*), finger millet (*Eleusine coracana*), foxtail millet (*Setaria italica*), proso millet (*Panicum miliaceum*), barnyard millet (*Echinochloa* spp), kodo millet (*Paspalum scrobiculatum*) and little millet (*Panicum sumatrense*) are grown (Muskan et al., 2025; Mukherjee et al., 2025). Ancient millets were initially grown for human consumption and were more popular than those that are today. Millets are a group of minor seeded grasses predominantly grown in specific regional areas. Millets are C4 plants and therefore possess a highly efficient photosynthetic system compared with other cereal crops such as wheat, barley, and rice. As a result, they are considered better adapted to withstand and cope with climate change (Venkatesh et al., 2023; Gao et al., 2025b). Despite their long agronomic history and nutritional attributes, millets have been largely overlooked in modern agriculture over the past century, as research and policy favored higher-yielding cereals such as maize, rice, and wheat (Shanker, 2024). However, rising concerns over global food insecurity, malnutrition and environmental degradation have renewed interest in leveraging millets unique strengths to sustainably enhance productivity and resilience across marginal agricultural environments (Patil et al., 2023). In addition, millets can be produced on infertile soil with a minimum water requirement, low fertilizer, insecticide and pesticide requirements (Pathak, 2023). As hardy, drought-tolerant grasses requiring minimal inputs, millets offer great promise for boosting yields and livelihoods across arid regions of Africa, Asia, and the Americas (Kheya et al., 2023). Millets are rich in protein, essential amino acids, vitamins and minerals, offering superior nutrition compared with staples such as rice and wheat (Tripathi et al., 2021). Moreover, Millets vast genetic and phenotypic diversity enables the breeding of climate-resilient, nutritionally enhanced cultivars suited to diverse environments and user needs (Patil et al., 2024). This diversity also strengthens adaptation to multiple stresses and market demands. Therefore, comparative benchmarking of millet genomics with major cereals such as rice, maize and wheat is essential to assess genomic maturity, translational readiness and infrastructure gaps for faster crop improvement (Jadhav et al., 2024). Rice leads to advanced reference genomes, pan-genomes, mutant libraries and strong databases, while maize excels in pan-genomics and functional genomics for complex traits. Wheat has also progressed remarkably with numerous reference assemblies and graph-based variation platforms despite its polyploid complexity (Naithani et al., 2023; N’Diaye et al., 2023).

In comparison, millets are still developing these genomic resources, but such benchmarking provides a clear pathway to accelerate precision breeding and efficient genetic improvement. In comparison, millet genomics is progressing rapidly but remains at an intermediate stage of genomic maturity. Several millet species, including pearl millet, foxtail millet, finger millet, and proso millet, now possess chromosome-scale reference genomes. In contrast, recent graph-based pan-genome analyses in pearl millet have successfully uncovered structural variants linked to heat adaptation and domestication-related traits (Basu and Parida, 2025; Zhang et al., 2025). However, unlike rice and maize, most millet species still lack large-scale mutant repositories, genotype-independent transformation systems, standardized gene-editing workflows, extensive expression atlases, and an integrated comparative knowledgebase. These limitations slow the transition from genome assembly to trait validation and deployment in breeding pipelines (Prabhakar et al., 2022). Therefore, a comparative cereal-genomics perspective highlights strong opportunities for technology transfer. However, species-specific challenges, such as recalcitrant transformation, weak phenomics infrastructure, limited diversity panels and fragmented databases still constrain the full potential of millet genomics (Kaur et al., 2023). Despite these limitations, the exceptional heat and drought resilience of millets makes them increasingly important as climate change expands arid regions worldwide, offering strong potential for food self-sufficiency (Timilsina et al., 2025).

Crops such as *S. bicolor*, *S. italica*, *P. miliaceum*, and *P. glaucum* possess comparatively more advanced genomic resources than minor millets such as *E. coracana*, *P. sumatrense*, and *P. scrobiculatum* (Meena et al., 2021; Rajasekaran and Francis, 2021; Satomura, 2025). This disparity primarily arises from differences in inherent genome characteristics, historical research prioritization, and economic relevance (Corpas et al., 2025). Among these, foxtail millet has emerged as a model system for studying C4 photosynthesis and grass genomics due to its relatively small, diploid, and less complex genome, along with a short life cycle and self-pollinating nature (Meena et al., 2024). The selection of such crops as model organisms led to concentrated funding and international research collaborations, giving them a significant head start in genomic advancements. In addition, the availability of well-structured genetic resources, including mapping populations, mutant libraries, and extensive germplasm collections, has further strengthened genomic research in major millets (Gomashe et al., 2025). Additionally, Institutional support from organizations such as the International Crops Research Institute for the Semi-Arid Tropics (ICRISAT) has also played a pivotal role in advancing genomic tools and breeding programs for these crops (Mwamahonje et al., 2024).

On the other hand, minor millets have remained relatively underutilized and geographically restricted, often cultivated by smallholder and indigenous farming communities. This limited global footprint has resulted in reduced research funding and slower development of genomic resources (Doggalli et al., 2024). Furthermore, biological complexity has posed additional challenges. For instance, finger millet exhibits an allotetraploid genome, which contains two distinct sets of chromosomes derived from different ancestral species. Such complexity historically made genome assembly and annotation difficult, particularly before the advent of advanced long-read sequencing technologies. Similar challenges have hindered genomic progress in little and kodo millets (Singh et al., 2025). Another key factor contributing to this disparity is the economic and agricultural importance of the crops. Major millets like sorghum and pearl millet are widely cultivated across continents and serve multiple purposes, including human consumption, animal feed, and biofuel production. Their large-scale cultivation and global market value have attracted both public and private investment, driving rapid advancements in genomics and breeding (Muskan et al., 2025). In contrast, minor millets often fall under the category of orphan crops, lacking strong commercial demand and institutional backing, which has historically limited their inclusion in large-scale genomic initiatives (Panthi et al., 2026; Chandra et al., 2024). With declining costs of genome sequencing technologies and increasing awareness of climate change and nutritional security, minor millets are gaining renewed scientific interest. Their inherent resilience to harsh environmental conditions and high nutritional value make them promising candidates for future agriculture (Kumar et al., 2024a; Sharma et al., 2026). Recent efforts, such as sequencing the finger millet genome and ongoing initiatives to develop genomic databases for little and kodo millets, indicate a positive shift toward bridging the genomic resource gap. This evolving research landscape suggests a promising future for integrating minor millets into mainstream genomic and breeding programs (Banshidhar et al., 2023). In this context, molecular breeding, integrating conventional breeding with advanced genomic tools, accelerating the precise selection and development of superior millet genotypes. MAS has proven particularly effective in tracking and pyramiding complex traits governed by multiple genes (Parihar and Shiwani, 2022). The identification and mapping of quantitative trait loci (QTLs) associated with key agronomic traits have greatly facilitated the development of improved cultivars through molecular marker-based breeding (Bhatia and Bajwa, 2022). In addition, the advent of genetic engineering and molecular breeding technologies have revolutionized millet improvement, enabling the development of high-yielding, nutrient-rich, and climate-resilient cultivars (Maurya et al., 2023).

Moreover, genetic engineering allows precise modification or introduction of beneficial genes to enhance agronomic performance and nutrient-use efficiency. Moreover, genome-editing tools such as clustered regularly interspaced short palindromic repeats (CRISPR/Cas) have further advanced millet breeding by enabling site-specific, multiplex gene edits without the insertion of foreign DNA, thereby improving stress tolerance, yield, and nutritional traits with fewer biosafety concerns (Numan et al., 2021). Concurrently, omics approaches such as genomics, transcriptomics, proteomics, and metabolomics have expanded insights into millet biology (Jadhav et al., 2024). High-quality reference genomes and pangenome analyses of species like *S. italica* and *P. glaucum* have revealed significant genetic diversity and novel alleles associated with abiotic stress tolerance and adaptability, laying a strong foundation for precision breeding and genetic improvement in millets (Maurya et al., 2022; He et al., 2023). To further accelerate the development of improved millet varieties, the integration of speed breeding, machine learning with advanced genomic tools has emerged as a promising approach. Speed breeding involves manipulating environmental parameters, such as photoperiod and temperature, to reduce generation time, thereby enabling multiple crop cycles within a single year (Potts et al., 2023). When combined with GS and molecular marker technologies, this strategy markedly shortens the breeding pipeline, facilitating the rapid release of superior cultivars (Kumar et al., 2024a). This integrated approach is particularly advantageous for millets, where breeding progress has historically been constrained by long generation durations and limited genetic resources (Vetriventhan et al., 2020; Yadav et al., 2021a; Kumar et al., 2024a). This comprehensive review synthesizes current knowledge on unlocking millets’ immense yet overlooked capacity to sustainably enhance productivity, nutrition, and resilience across the world’s arid agricultural regions.

## 2 Integration of QTL mapping and marker-assisted selection for stress tolerance

**Figure 1:**
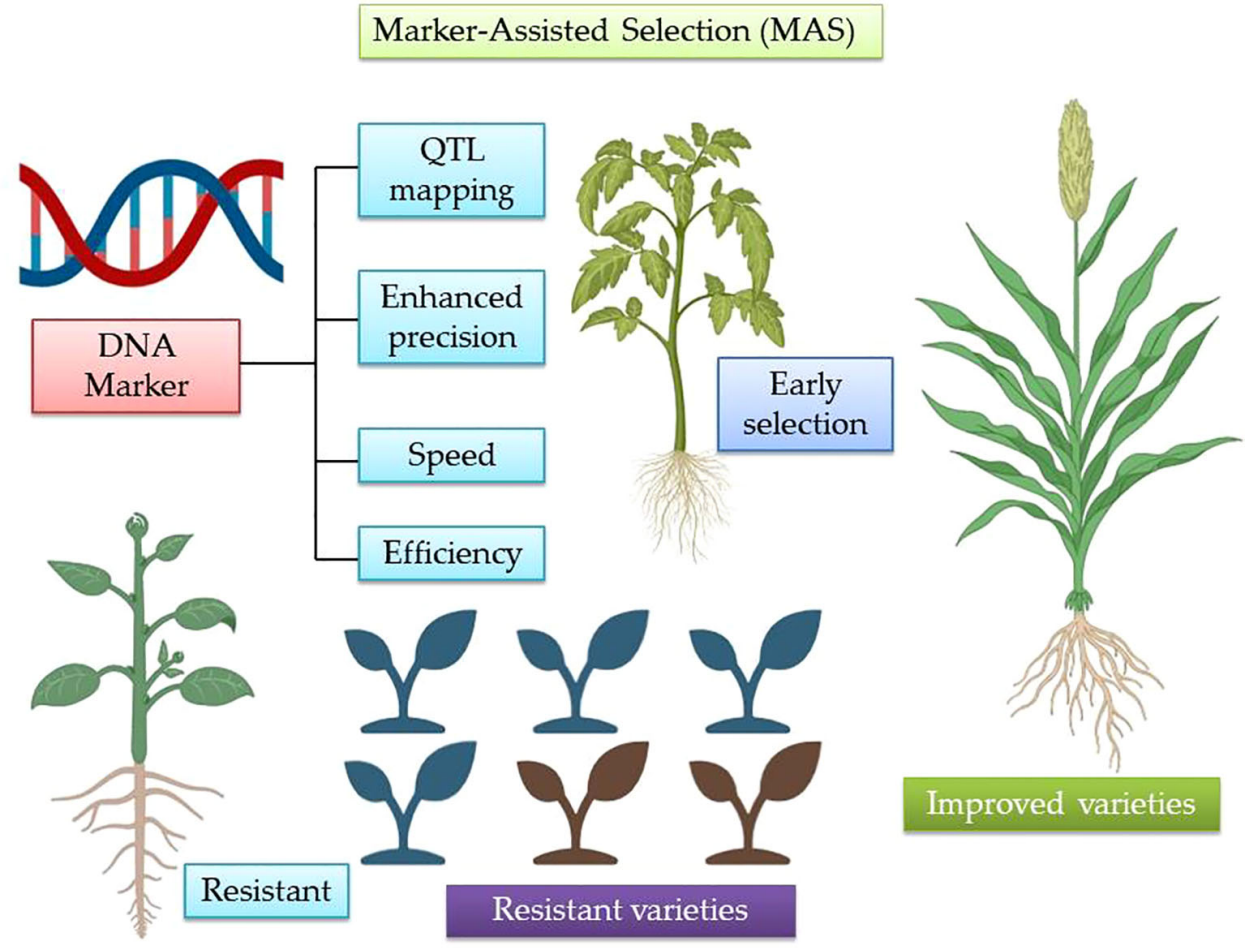
A schematic representation illustrating the pivotal role of marker-assisted breeding (MAB) in crop improvement, highlighting its efficiency in identifying, tracking and integrating desirable alleles for complex traits.

MAS has emerged as a powerful molecular breeding tool for improving tolerance to both biotic and abiotic stresses in crop plants (Kumar et al., 2022; Joshi et al., 2024). It involves the use of DNA markers closely linked to specific genes or QTLs governing desirable traits, enabling breeders to select superior genotypes at an early stage without relying solely on phenotypic evaluation (Kumar et al., 2025; Thakur et al., 2024). This approach significantly enhances the precision, speed, and efficiency of breeding programs, particularly for complex traits influenced by multiple genes and environmental interactions (Merrick et al., 2022). Traditional breeding methods often struggle to accurately identify and select for biotic and abiotic tolerance due to environmental variability and the complex inheritance of these traits (Lamichhane and Thapa, 2022). In contrast, MAS allows breeders to track and incorporate genes or QTLs associated with stress resistance (**Supplementary Table 1**), facilitating the development of resilient cultivars (**Figure 1**) (Lamichhane and Thapa, 2022).

In a study, for example, QTL mapping was conducted using 114 pearl millet progenies from a resistant × susceptible cross. Molecular mapping was performed using restriction fragment length polymorphism (RFLP) markers in an F_2_ population and disease incidence was evaluated in F_4_ families. Composite interval mapping (CIM) identified associations between the F_4_ family and marker genotypes. Despite environmental and methodological differences, two consistent QTLs for resistance were detected for the downy mildew across all screening environments (Jones et al., 2002). In another study, three biparental populations, each with 207 F_23_ lines, were evaluated in an alpha lattice design with two replications across two moisture stress environments. Composite interval mapping identified 105 QTLs, which were projected onto a consensus map to define 25 meta-QTLs associated with seven traits (Techale et al., 2022). In sorghum, major QTLs including Stg1, Stg2, Stg3, Stg4, Stg3A, and Stg3B have been mapped for stay green and high yield traits. These QTLs are widely used in marker-assisted backcrossing (MABC) to introgress stay-green alleles into senescence-prone sorghum varieties (Mwamahonje et al., 2021). Moreover, Genotyping of 168 F_7_ RILs from 81B-P6 × ICMP 451-P8 using 256 diversity array technologies (DArT) and 70 simple sequence repeat (SSR) markers generated a linkage map of 286 loci spanning 740.3 cM. A major rust resistance QTL on LG1 (LOD 27) explained 58% phenotypic variation, with two minor QTLs on LG4 and LG7. The LG1 QTL provides durable, slow-rusting resistance, effective in India for over two decades (Ambawat et al., 2016).

In a study, a high-quality 477 Mb foxtail millet genome assembly covering >97% of the genome was developed using *de novo* and reference guided approaches. Over 98% of genes were mapped to nine chromosomes, with improved annotation. Unique metabolic GO terms, growth-related regions on chromosome 9 and synteny with pearl millet and maize growth loci were identified, providing a valuable resource for genomics and breeding research (Tsai et al., 2016). Building on these genomic resources, subsequent diversity analyses across weedy, wild, landrace, and cultivated *Setaria* germplasm revealed substantial phenotypic and genetic variation. Inter-simple sequence repeat (ISSR) and SSR marker profiling confirmed this diversity, showing mean alleles per locus of 3.75 and 2.45, respectively, with consistent PIC values (0.34) and moderate-to-high Shannon’s indices. Furthermore, high gene flow (Nm > 1.0) and cluster analysis grouped the 12 accessions into two major clusters, in agreement with PCoA results, highlighting the rich genetic base available for future genomics-assisted breeding in *Setaria* (Palakurthi et al., 2023).

Extending similar diversity investigations to other millets, a subsequent study evaluated 61 barnyard millet genotypes from southern India using 10 morpho-nutritional traits and 51 expressed sequence tag (EST)-SSR markers, further broadening the understanding of genetic variation and trait potential across minor millet germplasm. Substantial variability was observed for key traits, including flowering (49.50-82.00 days), plant height (58.17-152.38 cm), grain yield (12.52-41.25 g), ferrous (Fe) (11.05-21.51 mg/100 g), and zinc (Zn) (2.46-5.91 mg/100 g). Out of 51 EST-SSR primers screened, 14 exhibited polymorphism and generated a total of 29 alleles, with PIC values ranging from 0.276 to 0.652 (mean 0.43). Additionally, cluster analysis grouped the 61 genotypes into 12 distinct clusters, revealing substantial genetic diversity among the studied materials. This occurred due to inherent genetic diversity among the genotypes and varying marker informativeness. Trait variation reflects genetic differences, whereas limited polymorphism in EST-SSR markers indicates uneven detection of allelic variation, leading to distinct genetic clustering (Manimekalai et al., 2018). In foxtail millet, superior F_3_ families from the Botok-10 × ICERI-6 crosses were selected using a weighted selection index with the SiDREB2 SNAP marker. Genotyping revealed 29 A/A, 121 A/G, and 28 G/G genotypes. Ten promising families, comprising seven A/G and three A/A lines, exhibited index values ranging from 0.84 to 3.76. Among these, the A/A lines B10I6-15-136, B10I6-15-161, and B10I6-15–70 were identified as transgressive segregants. This occurred due to genetic recombination and segregation in the F_3_ generation, which created new allele combinations. The SiDREB2 marker helped identify favorable alleles and the selection index captured superior trait combinations, resulting in high performing, transgressive segregants (Sari Butarbutar et al., 2024). An Ion S5 Next Generation Sequencer was used to sequence a 400 bp library of barnyard millet, generating a draft genome of 59, 67, 79, 933 bp across 11,39,481 contigs. A total of 46,157 SSRs were identified, with 41,591 sequences containing SSRs and 3,867 harboring multiple SSRs. Validation of 15 SSR markers across 30 accessions showed 66.54% polymorphism, with a mean PIC of 0.28 and SPI of 0.57. This occurred because high-throughput sequencing generated large amounts of genomic data, enabling the detection of numerous SSRs. However, the moderate polymorphism and PIC values reflect limited genetic variation among accessions and the relatively conserved nature of some SSR regions (Meniya et al., 2023).

Cross-genome transferability of 101 SSRs from finger millet and 26 from foxtail millet was assessed across eight millet species. Of these, 33 finger millet and 2 foxtail millet markers showed 100% transferability. Overall transferability ranged from 47.52-61.38% (finger millet) and 30.76-69.23% (foxtail millet), with EST-SSR markers exhibiting higher transferability than gSSR markers. This occurred due to conservation of genomic regions across millet species. EST-SSR markers showed higher transferability because they originate from expressed (coding) regions, which are more conserved than non-coding regions targeted by gSSR markers (Krishna et al., 2018). In proso millet, Principal Component Analysis (PCA) showed that the first four components explained 59.94% (normal) and 62.48% (salinity stress) of total variation, with genotypes grouped into three clusters under both conditions. Association analysis using 514 amplified fragment length polymorphism (AFLP) markers identified key marker-trait linkages: M14/E10–45 and M14/E10–60 with seed yield & M14/E10–45 and M14/E11–44 with forage yield under normal conditions, while M14/E10–14 and M14/E10–64 showed stable associations under salinity stress. This pattern arises from the complex genetic architecture of yield traits and genotype × environment interactions. PCA captured most of the variation through a few components, reflecting coordinated trait responses under both normal and stress conditions. Consistent clustering indicates stable genetic relationships among genotypes. The identified AFLP markers are likely linked to genomic regions controlling yield traits, while stress-specific associations reflect activation of adaptive genes under salinity, leading to stable marker-trait linkages across environments (Yazdizadeh et al., 2020). In proso millet, GBS-based mapping identified 5,621 high-quality SNPs across 160 accessions, with an average genetic distance of 0.268. In which, the race miliaceum showed the highest diversity, while ovatum showed the lowest. Population structure aligned with geographic origin and GWAS detected 40 marker trait associations (MTAs) (34 agronomic, 6 nutritional), with 20 located in annotated genes. Higher diversity in miliaceum reflects a broader genetic base and a longer domestication history, while lower diversity in ovatum suggests genetic narrowing. Population structure aligns with geographic origin due to adaptation and selection, and the identified MTAs indicate SNPs linked to genes controlling agronomic and nutritional traits (Vetriventhan et al., 2024).

MAS has evolved substantially from its early use as a simple tool for selecting single major genes to a sophisticated, integrative breeding strategy capable of addressing complex traits (Edukondalu et al., 2026). Another important development is the shift from single-marker selection to haplotype-based MAS. Rather than relying on individual markers, breeders now select favorable combinations of alleles across genomic regions, which better capture linkage and epistatic effects (Singh et al., 2026). This approach enhances genetic gain and reduces the risk of losing beneficial minor alleles during selection. In parallel, MAS is increasingly integrated with GS, where markers linked to major-effect QTLs are fixed through MAS while genome-wide markers capture the cumulative effects of numerous small-effect loci (Edukondalu et al., 2026). This complementary strategy accelerates breeding progress without compromising precision. Environment-responsive MAS represents another novel dimension, as marker-trait associations are now evaluated across diverse environments to account for genotype × environment interactions. Such approaches allow the identification of markers that are either stable across environments or specifically adaptive under stress conditions such as drought, heat or nutrient limitation, making MAS more relevant under climate change scenarios (Jadon et al., 2026). Moreover, marker-assisted pyramiding has advanced to enable the simultaneous accumulation of multiple resistance, stress-tolerance and nutritional quality traits within a single genetic background, supported by high-throughput and cost-effective marker systems.

## 3 Integrating GWAS and GS for accelerated trait mapping and genetic improvement in millet

Although biparental population-based QTL mapping has played a pivotal role in detecting loci linked to key agromorphological traits in crop plants, its effectiveness is constrained by several factors, including limited genetic resolution, small population size, restricted allelic diversity, environmental interactions and dependency on the number and nature of markers used. In contrast, GWAS have emerged as a more powerful alternative for dissecting complex traits (Saini et al., 2022). GWAS leverage the extensive ancestral recombination and genetic diversity of natural germplasm panels to identify precise associations between SNPs and causal variants (Harris et al., 2025). Unlike traditional linkage analysis, the smaller linkage disequilibrium (LD) blocks in diverse populations facilitate fine-mapping of target loci, allowing for their rapid integration into crop improvement programs (Scott et al., 2020; Desaint et al., 2023). In millets, GWAS has successfully identified genomic regions associated with key traits, including drought resilience, grain micronutrient content and disease resistance (Nakombo et al., 2025). Despite its precision, GWAS remains sensitive to population structure and primarily identifies common alleles with small effect sizes, often failing to capture rare variants or complex gene environment interactions. Consequently, GS is increasingly employed as a complementary strategy to address these limitations and enhance breeding efficiency (Xue and Cui, 2025). Additionally, GS uses phenotypic data and high-density marker profiles to predict breeding values, leveraging information from all markers to reduce bias and capture variance contributed by small effect QTLs (Kumar et al., 2024a). With the recent availability of the pearl millet reference genome, GS models can now be effectively integrated into breeding programs. By utilizing genome-wide marker derived genomic estimated breeding values, GS enables more accurate and efficient improvement of complex quantitative traits (Sehgal, 2016).

Several studies have demonstrated the effectiveness of GWAS and GS in crop improvement, particularly in millets and related cereals. In pearl millet, high-density genomic analysis identified over 1.45 million SNPs and 124,532 structural variants (SVs) across 242 inbred lines, with SV based models outperforming SNP-based models for genomic prediction, gBLUP showed the best performance for SVs, while Bayesian models were more suitable for SNPs (Yan et al., 2024). Similarly, GWAS using 67K SNPs in 222 accessions identified 218 significant SNPs associated with antioxidant activities, leading to the discovery of 18 candidate genes involved in key metabolic pathways (Yadav et al., 2021b). Genetic structure analysis using GBS-derived SNPs further revealed distinct gene pools associated with variation in flowering time (Diack et al., 2020). In addition, several MTAs have been reported for nutritional traits, including Fe and Zn content, with co-segregating SNPs and candidate genes linked to biofortification (Pujar et al., 2020). Large-scale GWAS in Pearl Millet inbred Germplasm Association Panel (PMiGAP) panel identified 544 significant associations for protein and amino acid traits, including pleiotropic loci and numerous candidate genes for nutritional improvement (Singh et al., 2024).

In sorghum, GWAS has identified 40 SNPs associated with key traits such as heading date, plant height, dry yield and phenolic content, with candidate genes like *FUT1*, *MAFB* and *PDHA1* playing important roles in phenology, yield and metabolite biosynthesis (Lee et al., 2023). Similarly, several quantitative trait nucleotides (QTNs) and candidate genes associated with flowering time and other morphological traits have been reported, highlighting the genetic basis of plant architecture (Behera et al., 2024; Wang et al., 2025a). High-density resequencing of global germplasm has further enabled the identification of significant QTLs for yield-related traits after accounting for population structure and kinship (Zhang et al., 2023). Moreover, multi-locus GWAS across diverse environments detected numerous stable QTNs associated with major agronomic traits, including flowering time, plant height and grain yield, demonstrating their potential for marker-assisted breeding in sorghum (Wondimu et al., 2023). In foxtail millet, GWAS identified significant SNPs associated with seed protein and amino acid composition, explaining moderate phenotypic variation, while genomic prediction showed accuracies ranging from low to moderate across traits (Zhao et al., 2024). Similarly, multiple marker-trait associations for mineral elements, including Fe, Zn, magnesium (Mg) and boron (B), have been reported, highlighting their potential for nutritional improvement (Jaiswal et al., 2019). In addition, several stable QTL regions were detected across environments and integration of synteny, gene expression and haplotype analyses enabled the identification of high confidence candidate genes for key agronomic traits (Liu et al., 2022). GWAS and GS have undergone substantial methodological advancements, transforming them from isolated marker-based tools into integrated, predictive breeding strategies (Ahmed et al., 2026). Novel GWAS approaches now move beyond single-locus analyses and instead emphasize multi-locus models that simultaneously evaluate the effects of numerous markers, thereby improving the detection of minor effect loci underlying complex quantitative traits (Kumar et al., 2025). The use of haplotype based GWAS has further enhanced biological relevance by capturing linkage disequilibrium blocks rather than individual SNPs, while pan-genome enabled GWAS allows the inclusion of structural variations and presence or absence variations that are particularly important in large and complex crop genomes (Kumbhakar et al., 2026). Additionally, GWAS models integrating genotype × environment (G × E) interactions have facilitated the identification of both stable and environment-specific loci, thereby providing critical insights for the development of climate-resilient cultivars. In parallel, GS has progressed from conventional GBLUP-based approaches to more advanced predictive frameworks incorporating deep learning, multi-trait prediction and environment specific modeling, further enhancing breeding precision and adaptability (Ghorbanzadeh et al., 2025). Modern GS approaches efficiently capture additive as well as non-additive genetic effects, including dominance and epistasis, thereby improving prediction accuracy for complex traits such as yield, stress tolerance and nutrient use efficiency (Liao et al., 2026). Reaction norm and genotype × environment aware GS models allow the prediction of genotype performance across diverse environments, supporting location-specific variety deployment. Furthermore, biologically informed GS models that utilize haplotypes, candidate genes or GWAS-weighted markers have improved long-term genetic gain while reducing computational complexity (Sidahmed et al., 2025). Although the GS framework in millets has benefited substantially from methodological advances established in sorghum and other major cereal systems, its translational relevance to millet breeding requires careful contextualization. Sorghum serves as a valuable comparative model because of its close phylogenetic relationship, partially conserved genome architecture and shared adaptation to arid and semi-arid environments, which collectively support the transferability of marker-trait prediction strategies (Prabhakar et al., 2022; Mwamahonje et al., 2024). Nevertheless, direct implementation of GS in millet breeding programs remains comparatively limited and is currently concentrated in a few species, particularly pearl millet, where conventional prediction models such as GBLUP and Bayesian regression approaches are increasingly being evaluated (Variath et al., 2022; Sinha et al., 2023). In contrast, the application of deep-learning enabled GS models in millets are still at an emerging stage, with most reported frameworks largely extrapolated from validated studies in major cereals rather than widely deployed in routine millet breeding pipelines (Wang et al., 2022a; Taha et al., 2025). Therefore, these advanced architectures should presently be interpreted as highly promising and transferable future opportunities rather than as fully established breeding tools in millet improvement.

## 4 Advances in millet cultivation through omics approaches

**Figure 2:**
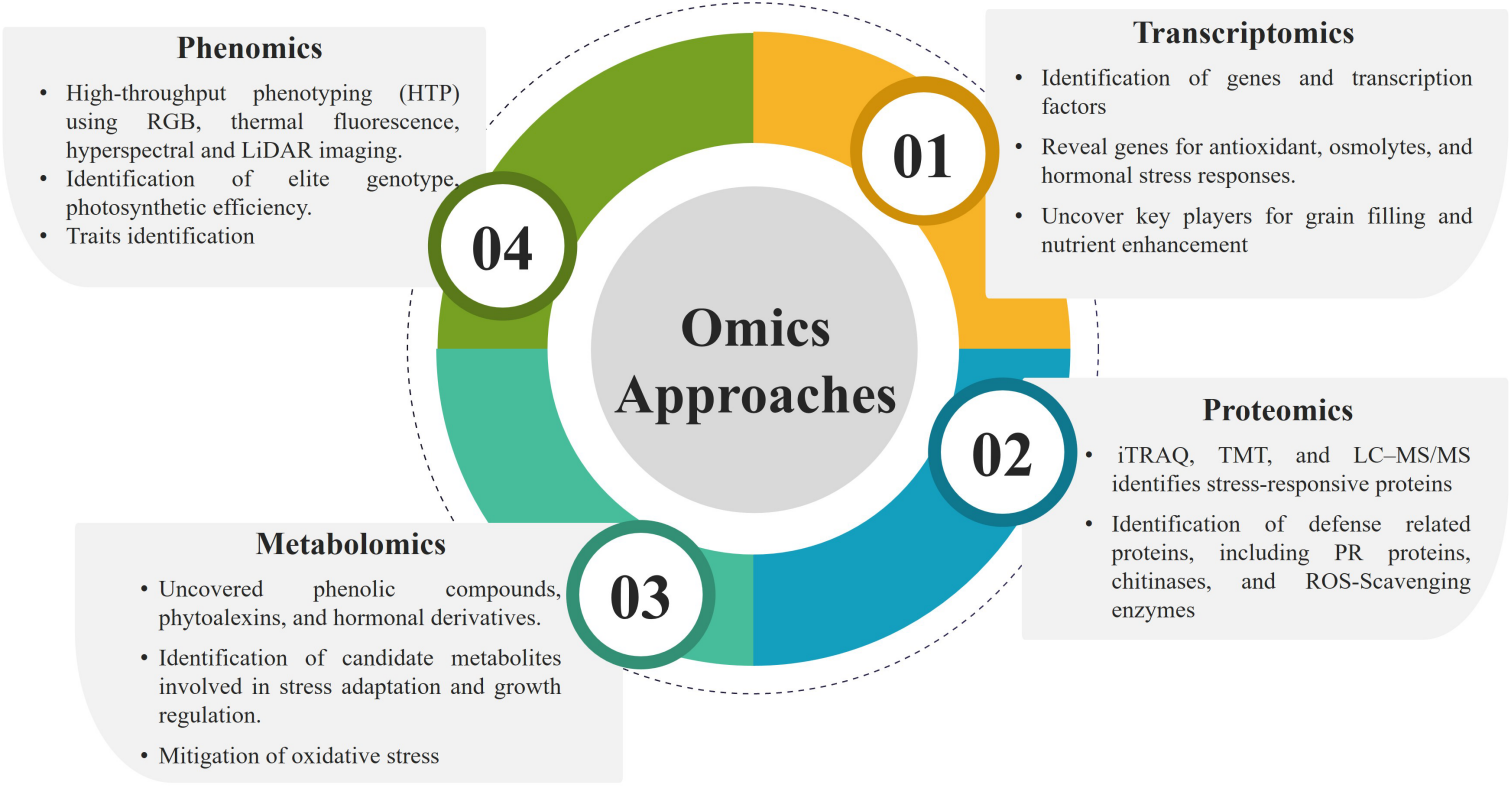
Schematic representation illustrating integrated omics approaches advancing genetic improvement and stress resilience in millets.

Omics technologies in millets encompass transcriptomics, proteomics, metabolomics and phenomics, enabling a comprehensive understanding of their genetic architecture, molecular regulation and physiological responses (**Figure 2**). In addition, key milestones in millet genetic resource development and genomic advancements have been incorporated to provide a clearer historical perspective, thereby strengthening the manuscript’s scientific relevance and continuity (**Table 1**). Hence, these integrative approaches facilitate the identification of stress-responsive genes, molecular markers and metabolic pathways, thereby accelerating genetic improvement and enhancing resilience, yield potential and nutritional quality in millet breeding programs.

**Table 1:** Key milestones in millet genetic resource development and genomic advancements.

Year	Milestone	Millet species	Significance	References
Pre-2000s	Germplasm Collection & CMS Discovery	Pearl Millet	Establishment of large germplasm banks (ICRISAT); Discovery of Cytoplasmic Male Sterility (CMS) enabling commercial hybrid breeding.	Yadav et al., 2024a
2008	First EST-SSR Markers	Pearl Millet	Development of 21 polymorphic EST-SSRs and 6 genomic SSRs used for genome mapping and marker-aided breeding for drought and yield.	Srivastava et al., 2020
2010	Reference Set Development	Pearl Millet	Development of a genotype-based reference set (300 accessions) for association mapping and allele mining.	Srivastava et al., 2020
2012	First Draft Genome Sequence	Foxtail Millet	First millet genome sequenced (~490 Mb); became the reference genome for comparative genomics in millets due to small size.	Sun et al., 2024
2014-2017	Whole Genome Sequencing (WGS)	Finger Millet	Draft genomes published (ML-365 and PR 202 lines); ~78–82% coverage; identified ~62k–85k genes despite complex allotetraploid nature.	Ajeesh et al., 2022
2017	Pearl Millet Reference Genome	Pearl Millet	High-quality reference genome of line Tift 23D2B1-P1-P5 released; estimated 38,579 genes; catalyzed functional genomics.	Yadav et al., 2024a
2017-2020	PMiGAP & GWAS Resources	Pearl Millet	PMiGAP; enabled GWAS using >32 million SNPs.	Srivastava et al., 2020
2020-2022	GS Models	Pearl Millet	Implementation of GS using whole-genome re-sequencing (WGRS), RAD, and tunable genotyping-by-sequencing (tGBS); multi-environment phenotyping for yield and stress tolerance prediction.	Srivastava et al., 2020
2023	Graph-Based Pan-Genome	Foxtail Millet	First graph-based pan-genome constructed; revealed extensive structural variations missed by short-read assemblies; enabled key gene mining.	Liang and Han, 2024
2023	Pearl Millet Pan-Genome	Pearl Millet	Graph-based pan-genome of 10 diverse lines; discovered 424,085 nonredundant SVs (74.7% presence or absence variations) linked to heat/drought adaptation.	Raza et al., 2023
2024	Genomic SSR Database (GSMDB)	Multiple Millets	Launch of centralized database for genomic SSRs across millet species, facilitating marker development and comparative genomics.	Kumar et al., 2024b

### 4.1 Role of transcriptomics in understanding millet gene expression

Transcriptomics, the comprehensive study of RNA transcripts expressed by an organism, has emerged as a powerful tool for understanding gene expression and regulatory mechanisms in millets (Dhawi, 2024). Owing to their exceptional resilience, nutritional richness and adaptability to marginal environments, millets have become model crops for transcriptomic research aimed at elucidating stress tolerance and productivity traits (Muruganantham et al., 2025). Transcriptomic studies have revealed that stress tolerance in millets is governed by complex gene networks involving antioxidant enzymes, osmolyte biosynthesis pathways and regulatory proteins such as DREB, WRKY, NAC and MYB transcription factors (Prusty et al., 2024). For instance, in pearl millet, genes encoding heat shock proteins (HSPs), late embryogenesis abundant (LEA) proteins and reactive oxygen species (ROS) scavenging enzymes are significantly upregulated under abiotic stress conditions which highlighting their role in cellular protection and stress mitigation (Singh et al., 2024). Similarly, transcriptome profiling in finger millet under drought and salinity stress has identified genes associated with ion transporters, abscisic acid (ABA) signaling and photosynthetic stability, which contribute to its superior stress resilience compared to other cereals (Rahman et al., 2014). Millets’ resilience is driven by a coordinated molecular defense that maintains cellular homeostasis under extreme stress. This tolerance is justified by the upregulation of HSPs and LEA proteins, which act as chaperones to prevent protein denaturation and maintain structural integrity (Singh et al., 2024; Prusty et al., 2024). Simultaneously, robust ROS-scavenging systems neutralize oxidative damage, while ABA mediated signaling and ion transporters regulate stomatal conductance & Na^+^ and K^+^ balance (Liu et al., 2022). Supported by the efficient C4 pathway, these integrated mechanisms enable millets to sustain productivity in marginal environments where conventional cereals fail (Vivitha and Vijayalakshmi, 2015). Moreover, transcriptomic analysis identified 8,887 and 12,249 differentially expressed genes (DEGs) in Yugu2 and An04, respectively, with 3,149 DEGs shared between the two varieties (Pan et al., 2020). Transcriptome analysis of three foxtail millet cultivars identified 2,954, 1,531 and 2,344 drought-responsive DEGs in Jigu39, Jingu21 and Longgu16, respectively. Enrichment in photosynthesis and metabolic pathways was observed, with 46 genes, including 32 novel ones, associated with drought tolerance (Guo et al., 2023). Variations in gene regulation and metabolic adjustments among cultivars lead to differential expression patterns that reflect their distinct levels of drought tolerance (Guo et al., 2023; Gao et al., 2025a). Quantitative profiling of a foxtail millet RIL population revealed five fold higher grain flavonoid content in the high-flavonoid (HF) group compared to the low-flavonoid (LF) group. Transcriptomic analyses identified key regulatory networks, with the HF and LF groups exhibiting distinct expression patterns of flavonoid biosynthesis related genes (Ye et al., 2025). Beyond stress responses, transcriptomics has also provided insight into developmental and nutritional regulation in millets.

Using high-throughput RNA sequencing (RNA-seq) technologies, researchers have identified DEGs, transcription factors and metabolic pathways involved in responses to drought, salinity, heat and nutrient deficiency (Bashir et al., 2019). For example, RNA-seq profiling in pearl millet infected with *Sclerospora graminicola* demonstrated the activation of genes linked to salicylic acid, jasmonic acid and ethylene signaling pathways, which are central to systemic acquired resistance (SAR) and induced systemic resistance (ISR) (Kulkarni et al., 2016). Similarly, in foxtail millet infected with blast fungus, transcriptomic data highlighted the induction of R genes (resistance genes), WRKY transcription factors and MAP kinase signaling components that orchestrate early defense responses (Sun et al., 2024). Moreover, RNA-seq analyses during grain filling and panicle development have revealed key genes controlling starch synthesis, protein accumulation and micronutrient enrichment (Wang et al., 2020).

**Figure 3:**
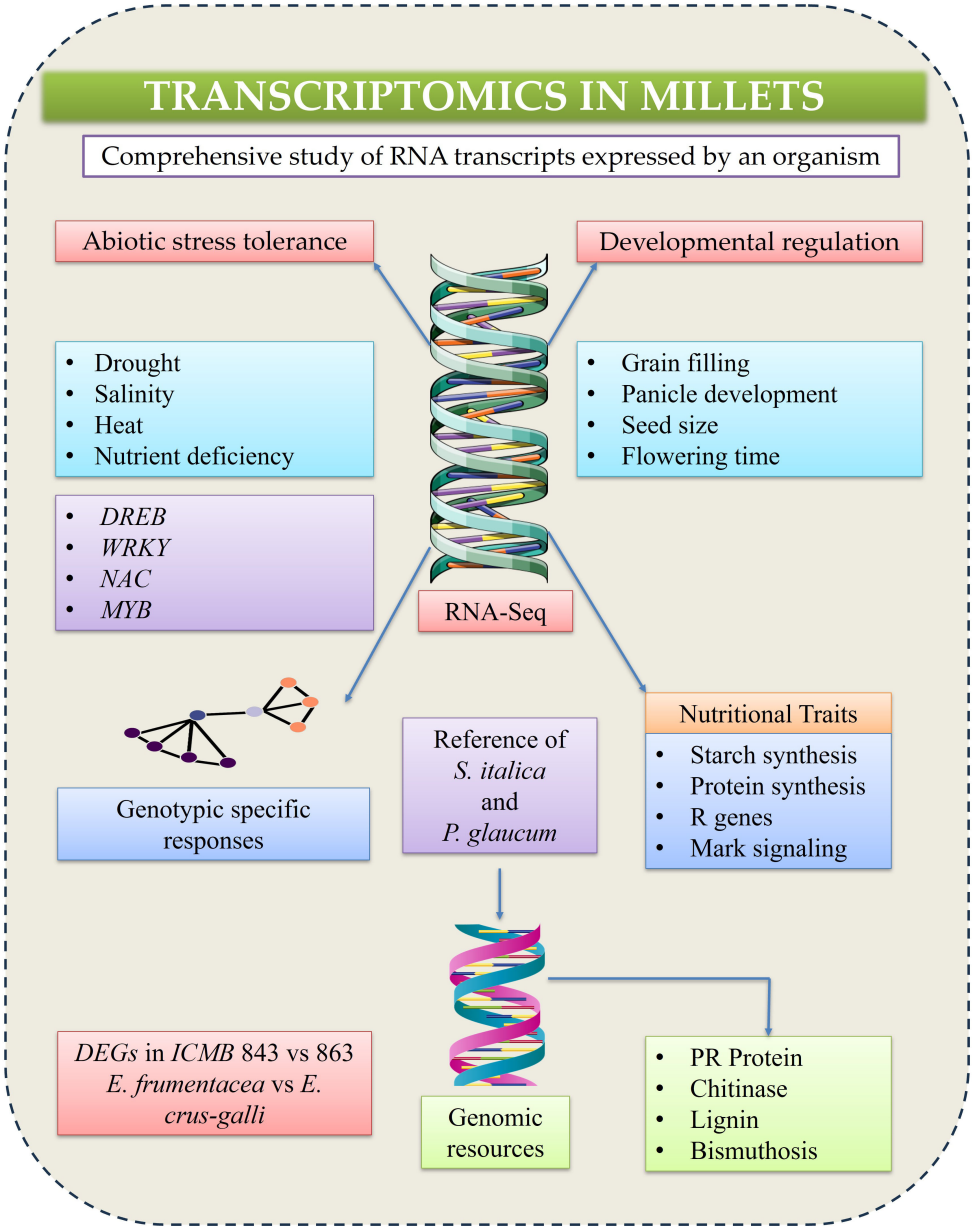
A schematic representation illustrates transcriptomics approach used to analyze gene expression patterns.

For example, RNA-seq analysis across five grain-filling stages in foxtail millet identified 11,399 DEGs, including 902 transcription factors, reflecting a metabolic shift from vegetative growth to reproductive sink strength. Functional and temporal profiling revealed key candidates potentially regulating the grain-filling process (Wang et al., 2020). Furthermore, developmental transcriptomics has uncovered the genetic basis of traits such as seed size, inflorescence structure and flowering time, which are important for yield improvement (Wang et al., 2023). The availability of high-quality reference genomes for *S. italica* and *P. glaucum* has facilitated accurate mapping of transcriptomic reads and identification of gene families involved in stress adaptation (**Figure 3**) (He et al., 2024). Additionally, transcriptome analyses under pathogen infection have revealed significant upregulation of defense related genes, including pathogenesis related (PR) proteins, chitinases, glucanases and key enzymes involved in phenylpropanoid and lignin biosynthesis, contributing to reinforcement of plant structural and biochemical barriers (Wang et al., 2022b).

In a study, root transcriptome analysis of two foxtail millet lines under control and drought conditions using the Illumina HiSeq platform generated 139.1 million reads. Mapping to the reference genome identified drought-responsive DEGs, with 6,799 and 1,253 genes detected in ICMB 843 and ICMB 863, respectively, highlighting genotype-specific transcriptional responses (Dudhate et al., 2018). The difference in DEGs arises from genotype-specific drought responses. ICMB 843 shows extensive transcriptional reprogramming, whereas ICMB 863 exhibits a more limited or stable response, reflecting differences in genetic background and stress adaptation mechanisms (lv et al., 2018). In foxtail millet, 2,792 transcription factors (TFs) and 1,223 transcriptional regulators (TRs) were identified, of which 318 TFs and 149 TRs responded to heat stress and 315 TFs and 128 TRs to drought stress (Sun et al., 2020). Moreover, a total of 11,627 DEGs were identified in foxtail millet, linked to pathways including hormone and MAPK signaling. Upregulation of genes related to ubiquitin mediated proteolysis and phenylpropanoid biosynthesis in the drought-tolerant line suggests their role in stress adaptation (Shinde et al., 2018). This result from stronger activation of stress responsive pathways in the tolerant line, leading to upregulation of genes involved in signaling, protein turnover and protective metabolic processes that enhance drought adaptation (Zhang et al., 2024; Gao et al., 2025a). Additionally, in kodo millet, a total of 239.1 million clean reads were generated, revealing 9,201, 9,814 and 2,346 DEGs in the 0 h vs 3 h, 0 h vs 6 h and 3 h vs 6 h comparisons, respectively. These DEGs were mainly associated with key pathways, including hormone metabolism and signaling, antioxidant defense, photosynthesis and cellular metabolism, and were further validated by qRT-PCR (Suresh et al., 2022). A total of 1,790 transcripts were differentially expressed under drought stress. Key signaling genes, including *serine/threonine protein phosphatase 2A* (*PP2A*), *calcineurin B-like interacting protein kinase 31* (*CIPK31*), *farnesyl pyrophosphate synthase* (*FPS*) and *signal recognition particle receptor α* (*SRPRα*) were activated, indicating their roles in drought perception and adaptation in foxtail millet (Parvathi et al., 2019). Moreover, reverse Northern analysis identified 327 differentially expressed transcripts, of which 86 showed ≥1.7-fold induction. qRT-PCR validation of selected genes revealed genotype-specific expression patterns between tolerant (Prasad) and sensitive (Lepakshi) cultivars under dehydration stress (Lata et al., 2010). This arises from genotype-specific regulatory differences. The tolerant and sensitive cultivars differ in gene regulation and stress signaling efficiency, leading to distinct transcriptional responses under dehydration stress (Gao et al., 2025a) Jayakodi et al., 2019 reported the first comprehensive transcriptomes of *Echinochloa frumentacea* and its wild relative *E. crus-galli*. The *E. frumentacea* transcriptome comprised 97,065 transcripts, including 65,276 protein-coding and 31,789 long non-coding RNA (lncRNA) transcripts, with over 90% of these transcripts functionally annotated. The *E. crus-galli* transcriptome contained 93,725 transcripts, including 68,480 protein-coding transcripts, with 89% successfully annotated (Jayakodi et al., 2019). The differences are due to genomic complexity and species-specific gene composition. Variation in transcript number and annotation efficiency reflects differences in gene content, expression patterns and the availability of reference databases for functional annotation between the two species (Maharajan et al., 2024).

Additionally, one promising advancement is single-cell and spatial transcriptomics, which enables the resolution of cell-type-specific and tissue-specific gene expression during critical developmental stages or under stress conditions, allowing precise identification of regulatory hubs controlling yield and stress resilience (Rautela et al., 2026). The integration of time-series transcriptomics with phenological stages enables the capture of dynamic transcriptional reprogramming, thereby enhancing our understanding of temporal gene networks involved in adaptation (Yi et al., 2022). Another novel dimension is the use of pan-transcriptomics, which explores expression diversity across multiple genotypes, landraces and wild relatives to uncover rare or condition-specific transcripts associated with stress tolerance and agronomic traits (Bhatti et al., 2020).

However, environment-responsive transcriptomics, combined with high-throughput phenotyping (HTP) and multi-omics data, enables the identification of genes exhibiting stable or plastic expression across diverse environments, thereby supporting climate-smart crop breeding. However, transcriptomics also has several limitations that restrict its direct application in breeding programs (Zhou et al., 2022; Ravichandran et al., 2025). Gene expression profiles are highly dynamic and context-dependent, varying across developmental stages, tissues and environmental conditions, which can reduce reproducibility and complicate the identification of stable candidate genes (Thera et al., 2026). Moreover, transcript abundance does not always correlate with protein levels or functional activity due to post-transcriptional, translational and post-translational regulation. Technical challenges, such as RNA instability, biases in library preparation and difficulties in capturing low abundance transcripts, further limit accuracy (Kumar et al., 2025; Li et al., 2026). On the other hand, proteomics provides complementary advantages by offering a more direct representation of the functional molecules within the cell (Jadhav et al., 2024). Since proteins are the primary effectors of biological processes, proteomics enables the identification of key enzymes, regulatory proteins and stress-responsive factors that are directly linked to phenotype expression. It also facilitates the detection of post-translational modifications, such as phosphorylation and glycosylation, which are critical for protein activity and cannot be inferred from transcriptomic data alone (Sharma et al., 2023). Furthermore, proteomics can improve the understanding of metabolic pathways, protein-protein interactions, and stress adaptation mechanisms under varying environmental conditions. When integrated with transcriptomics, and proteomics enhances the reliability of candidate gene identification and provides a more holistic view of the molecular basis of complex traits (Satrio et al., 2024).

### 4.2 Role of proteomics in understanding millet physiology and adaptation

Proteomics, the large-scale study of proteins and their functions has emerged as a crucial tool for unraveling the complex molecular mechanisms that regulate physiological and biochemical processes in plants (Satrio et al., 2024). Since proteins act as the primary executors of genetic information, proteomic approaches offer a functional perspective that extends beyond the transcriptome or genome, particularly under dynamic environmental conditions (Aslam et al., 2024). Proteomic analysis in millets employs a variety of high-throughput platforms and analytical strategies. Traditional two-dimensional gel electrophoresis (2-DE) coupled with mass spectrometry (MS) remains a foundational approach for identifying differentially expressed proteins (DEPs) under specific treatments (Singh et al., 2024). However, recent advances in label-free quantification, isobaric tags for relative and absolute quantitation (iTRAQ) and tandem mass tag (TMT) based methods have improved sensitivity and throughput (Sivanich et al., 2022; Devanna et al., 2023). For instance, comparative proteomics using 2-DE and MALDI-TOF has been employed in proso millet to study differential protein expression under drought and salinity stress (Mythri et al., 2024).

Additionally, sub-proteomic studies targeting chloroplast, mitochondrial or nuclear proteomes have been conducted to understand compartment-specific responses (Figueiredo et al., 2024). Bioinformatics tools, such as MapMan, STRING and KEGG pathway analysis are extensively utilized to categorize identified proteins into functional groups, including photosynthesis, energy metabolism, stress defense and signal transduction pathways (Karami et al., 2023). Drought-responsive proteomic analysis in finger millet revealed the upregulation of antioxidant enzymes, such as superoxide dismutase, catalase and peroxidases, as well as LEA proteins, dehydrins and HSPs, which aid in maintaining cellular homeostasis (Li et al., 2021). Integrated transcriptome and proteome analysis identified 1,305, 1,093 and 607 DEGs-DEPs in the N1-N2 (control vs. drought), N1-N3 (control vs. rehydration) and N2-N3 (drought vs. rehydration) comparison groups, respectively. GO and KEGG analyses revealed 80, 49, and 59 drought stress associated DEGs and DEPs in these groups, respectively (Li et al., 2021). Moreover, salinity-stressed pearl millet seedlings showed increased accumulation of vacuolar ATPases, Na^+^/H^+^ antiporters and compatible solute-related enzymes, including proline dehydrogenase, indicating that ion homeostasis and osmotic adjustment are key adaptive mechanisms under salt stress (Karthik et al., 2024). Proteomic analysis identified 4,015 proteins, with 610 and 276 differentially expressed in hybrid Zhangzagu10 relative to its female and male parents, respectively. Pathways related to metabolism, carbon fixation, and photosynthesis were significantly enriched. qRT-PCR further validated higher expression of selected genes in Zhangzagu10, supporting its superior physiological performance (Weng et al., 2020). These proteomic insights provide targets for marker-assisted breeding and transgenic development of salt-tolerant varieties (Banshidhar et al., 2023).

Heat stress–induced proteomic analyses in foxtail millet and barnyard millet demonstrated increased accumulation of small HSPs, chaperonins, and other protein-folding and stabilizing factors, underscoring their pivotal roles in preserving proteome integrity and cellular homeostasis under elevated temperatures (Ali et al., 2025; Kumar et al., 2025). Additionally, antioxidant enzymes were upregulated to reduce oxidative damage. At the same time, proteins involved in photosynthetic electron transport and Calvin cycle pathways remained highly heat-sensitive, underscoring the need to safeguard photosynthetic machinery under global warming conditions (Perveen et al., 2025). In finger millet, calcium-binding and storage proteins underpin high calcium accumulation, while phosphorus and nitrogen starvation proteomes reveal induction of phosphatases, nitrate reductases and amino acid metabolism enzymes that enhance nutrient use efficiency (Sharma et al., 2022; Prathap et al., 2023). Defense-related proteins, such as pathogenesis-related (PR) proteins, chitinases, β-1,3-glucanases and thaumatin-like proteins, have been identified during pathogen infection (Sharma et al., 2021b).

In pearl millet, infection with *Sclerospora graminicola* (downy mildew pathogen) triggered the expression of PR proteins, ROS scavenging enzymes and cell-wall modifying proteins (Meena et al., 2022; Han et al., 2025). Similarly, proteomic profiling of foxtail millet challenged with *Ustilago* spp. revealed upregulation of signaling proteins involved in MAP kinase cascades and defense hormone pathways, indicating (SAR) (Hao et al., 2020). Root, leaf and seed tissues were analyzed, leading to the identification and quantification of 2,281 proteins in total. Among these, leaf tissue exhibited the highest number of significant changes, followed by roots and seeds (Patil et al., 2023). Leaves are the most metabolically active tissues, serving as the center of photosynthesis, energy production and environmental sensing. Their highly dynamic physiological role leads to stronger protein reprogramming than in roots and seeds. Roots show more specialized changes related to nutrient uptake and signaling, while seeds remain comparatively stable due to their storage and maturation functions. Hence, leaves exhibited the highest number of significant proteomic changes. Additionally, elevated levels of root proteins associated with cell wall synthesis, lipid metabolism, secondary metabolism and signaling pathways, along with increased root length, suggest disrupted shoot-root communication under drought stress (Ghatak et al., 2016). In a study, total protein extracts from two-day-old pearl millet seedlings, collected six hours after pathogen inoculation, were resolved by 2-DE using IPG strips (pI 5–8). PD Quest analysis identified 210 protein spots, with 21 upregulated and 14 downregulated, indicating clear proteomic responses to pathogen infection (Hadimani et al., 2023). Pathogen infection rapidly activated early defense signaling in pearl millet seedlings, causing rapid proteome reprogramming. Defense and stress-related proteins were upregulated, while normal growth associated proteins were downregulated, reflecting a rapid shift from growth to protection (Shah et al., 2024). A total of 67 proteins showed differential abundance under salinity stress, highlighting a coordinated stress response. Key salt-responsive proteins included granule bound starch synthase 1, α-amylase, SPO11, flavonoid 3′-monooxygenase, ethylene receptor 4, transcription initiation factor Y 11g and auxin responsive protein IAA16. STRING analysis linked these proteins to a broader interaction network of 171 proteins, suggesting their collective role in salinity tolerance (Somasundaram et al., 2022). Salinity disrupted ion and water balance, so the plant altered proteins for energy, signaling and defense to tolerate stress (Arif et al., 2020).

Moreover, grain protein content (GPC) is a key indicator of foxtail millet’s nutritional value and contributes to drought tolerance. GPC is involved in multiple metabolic pathways, including starch and sucrose metabolism, glycolysis or gluconeogenesis, amino acid biosynthesis, detoxification and defense, protein degradation, the tricarboxylic acid (TCA) cycle, protein synthesis, energy metabolism, transport, the pentose phosphate pathway and signal transduction (Li et al., 2019). Moreover, proteomic analysis of mature seeds across three drought levels in Longgu6 and Huangjinmiao (HJM) identified DEPs, especially under moderate stress. In HJM, upregulated protein biosynthesis and drought-responsive pathways likely enhanced GPC (Xu et al., 2020). Fourteen CEP genes were identified in foxtail millet. All SiCEPs showed inducible expression under abiotic stresses and phytohormone treatments. In response to ABA, increasing SiCEP3 expression enhanced seedling growth inhibition and elevated ABA accumulation (Zhang et al., 2021). Selenium treatment altered the foxtail millet proteome, with 123 DEPs identified. Foliar spray of sodium selenite (Na_2_SeO_3_) at key growth stages increased selenium accumulation. Conserved C-terminally encoded peptides (CEPs) regulate growth and may inhibit plant development (Liang et al., 2020). Further, quantitative proteomic analysis identified 2,474 proteins in foxtail millet seedlings exposed to drought stress, of which 321 showed significant differential expressions, including 252 upregulated and 69 downregulated proteins. Gene Ontology annotation revealed that these proteins were involved in stress and defense responses, photosynthesis, carbon metabolism, ROS scavenging, protein synthesis and related functional categories (Pan et al., 2018).

The advancements of proteomics are shifting the focus from descriptive protein catalogs to functional, predictive and field-relevant applications that directly support breeding and stress-resilient agriculture. One emerging approach is spatio-temporal and single-cell proteomics, which enables protein-level resolution in specific tissues, cell types or developmental stages under normal and stress conditions, revealing post-transcriptional regulation that cannot be captured by transcriptomics alone (Schippers and Mueller, 2010). Another major advancement is quantitative and phospho-proteomics guided trait dissection, where protein abundance, turnover and post-translational modifications (PTMs) such as phosphorylation, ubiquitination and acetylation are mapped to identify signaling networks controlling stress tolerance, nutrient use efficiency and yield stability (Pal and Kundu, 2024). The integration of proteome-wide association studies (PWAS) with GWAS and QTL mapping represents a novel strategy to link protein variation directly with phenotypic traits, improving causal gene and pathway identification (Brandes et al., 2020). The application of environment-responsive and field-based proteomics, including stress memory and priming studies allow identification of proteins that confer resilience under fluctuating environments, which is highly relevant for climate-smart crop improvement (Moskal et al., 2025).

Importantly, the use of proteomics derived functional markers, including protein isoforms and PTM signatures, in combination with GS and genome editing, provides a novel translational route from discovery to breeding (Jha et al., 2025). However, proteomics also presents several limitations that may restrict its widespread application in crop improvement programs. Protein extraction and quantification are technically challenging, particularly for low abundance and membrane bound or highly hydrophobic proteins (Idowu et al., 2025). The proteome is highly dynamic and varies with tissue type, developmental stage and environmental conditions, which can affect reproducibility and comparability across studies. In addition, current proteomic platforms often provide limited coverage of the full proteome and the detection of rare proteins or subtle PTM changes remains difficult (Ceasar et al., 2024). In contrast, metabolomics offers several complementary advantages by capturing the end products of cellular processes, thereby providing a direct link to phenotype (Mazumder et al., 2024). Metabolites reflect the integrated outputs of gene expression, protein activity and environmental interactions, making metabolomics particularly valuable for understanding complex traits such as stress tolerance, nutritional quality and yield. It enables the identification of key metabolic biomarkers and pathways associated with adaptive responses under diverse environmental conditions (Mane et al., 2024). Moreover, metabolomic profiling can facilitate rapid phenotyping and the discovery of trait-associated metabolites that can serve as selection indices in breeding programs. When integrated with genomics, transcriptomics and proteomics, metabolomics enhances systems-level understanding and supporting the identification of robust, functionally validated targets for climate-resilient crop improvement.

### 4.3 Role of metabolomics in understanding millet metabolism and resilience

Metabolomics, the large-scale study of metabolites provides a functional readout of a plant’s physiological state (Shen et al., 2023). Because metabolites directly reflect biochemical activities and environmental interactions, metabolomics serves as a critical bridge linking genomic information to phenotypic expression (Sharma et al., 2021a). Metabolomic studies in millets have employed both targeted and untargeted approaches to characterize a wide range of metabolites involved in stress adaptation, growth regulation and nutritional quality (Mazumder et al., 2024). Targeted metabolomics focuses on specific classes of metabolites such as amino acids, sugars or flavonoids. In contrast, untargeted metabolomics aims to capture a broad profile of all detectable compounds to identify novel biomarkers and pathways (Azlan et al., 2026). The techniques, complemented by bioinformatics tools and metabolic databases such as KEGG and MetaboAnalyst, have facilitated the mapping of metabolic pathways and interpretation of biochemical alterations associated with stress tolerance and nutritional enhancement (Zhang et al., 2024). Drought stress, for instance, induces a significant accumulation of osmoprotectants, including proline, glycine betaine, sugars such as trehalose and sucrose and polyols like mannitol and sorbitol (Mishra et al., 2022). These metabolites function in osmotic adjustment, membrane stabilization and the detoxification of ROS (Dorion et al., 2021). Studies on foxtail millet and finger millet have revealed the upregulation of antioxidant compounds, including ascorbate, phenolics and flavonoids, during drought stress, highlighting the activation of oxidative defense pathways (Amoah et al., 2023). Moreover, intermediates of the TCA cycle such as malate and citrate increase under stress, suggesting an adaptive redirection of energy metabolism (Zhang and Fernie, 2023). For example, in pearl millet infected with *S. graminicola*, metabolic profiling revealed increased production of phenolic compounds, phytoalexins, and lignin precursors, all of which strengthen cell walls and provide antimicrobial defense (Sun et al., 2022).

Similarly, infection-induced accumulation of salicylic acid and jasmonic acid derivatives suggests hormonal cross-talks in defense regulation. In foxtail millet challenged by *Ustilago* species, elevated levels of flavonoids, terpenoids and antioxidants were observed, reflecting activation of multiple defense pathways (Sun et al., 2022). Such metabolomic signatures serve as potential biomarkers for identifying resistant genotypes and understanding host-pathogen interactions at the biochemical level (Mazumdar et al., 2023). Primary metabolite analyses have revealed high levels of essential amino acids, complex carbohydrates and unsaturated fatty acids that contribute to the superior nutritional and health-promoting properties of millets (Samtiya et al., 2023; Sabuz et al., 2023). Secondary metabolite profiling has identified a diverse range of phenolic acids, flavonoids, tannins and lignans that exhibit antioxidants, anti-inflammatory and antidiabetic properties (Sukhikh et al., 2023; Pujari and Hoskeri, 2022). For instance, finger millet is particularly rich in ferulic acid, gallic acid and catechins, while foxtail millet contains abundant quercetin and apigenin derivatives (Zhang et al., 2021). Comparative metabolomic analyses across different millet species and varieties have also highlighted metabolic diversity related to grain pigmentation, mineral content and antioxidant capacity, providing useful biochemical markers for biofortification and the development of functional foods (Yadav et al., 2021; Li et al., 2021).

Moreover, a total of 62 volatile compounds in foxtail millet were quantified using simultaneous distillation-extraction (SDE) followed by GC-MS analysis. The major components included 18 aldehydes, six alcohols, nine ketones, five acids, 10 hydrocarbons, 10 benzene derivatives and four other compounds. Among 30 odor-active compounds, 18 had odor activity values (OAV>1), including nonanal, (Z)-2-nonenal, (E,E)-2,4-nonadienal and naphthalene dibutyl phthalate. Partial least squares discriminant analysis (PLS-DA) identified minerals and aroma compounds with VIP>1 as key markers distinguishing foxtail millet varieties (Li et al., 2021). In response to salinity, 8,887 and 12,249 DEGs were identified in Yugu2 and An04, respectively, with 3,149 shared between the two varieties. GO and KEGG analyses showed that salinity-responsive genes in Yugu2 were predominantly enriched in ion transport, redox homeostasis, phytohormone metabolism, signaling, and secondary metabolism pathways (Pan et al., 2020). Quantitative profiling of a foxtail millet RIL population revealed five-fold higher grain flavonoid content in the high-flavonoid (HF) group compared to the low-flavonoid (LF) group. Integrated metabolomic and transcriptomic analyses identified ten flavonoids significantly enriched in HF lines and highlighted O-hydroxycinnamoyl transferase (HCT), phenylalanine ammonia-lyase (PAL) and phenylalanine/tyrosine ammonia-lyase (PTAL) genes as key regulators of differential flavonoid accumulation (Ye et al., 2025).

Some new ideas in metabolomics are redefining breeding by directly targeting the biochemical phenotype, which represents the closest link between genotype, environment and agronomic performance. A key emerging approach is spatio-temporal and tissue-specific metabolomics, enabling precise mapping of metabolites in specific organs, developmental stages or stress-responsive tissues to identify metabolic checkpoints controlling yield, quality and stress adaptation (Khan, 2025). Another innovative strategy is metabolome-wide association studies (mGWAS) and metabolic QTL (mQTL) mapping, where natural variation in metabolite abundance is directly associated with complex traits such as drought tolerance, nutrient use efficiency and disease resistance (Raza, 2022). This is further strengthened by metabolic biomarkers and signatures, which can be used as early-stage selection tools in breeding programs, even before phenotypic traits are fully expressed (Su et al., 2025). The integration of environment-responsive and stress-memory metabolomics is a novel concept, capturing metabolite plasticity and priming effects under recurrent or combined stresses, which is crucial for climate-resilient crop improvement (Talarico et al., 2025).

Additionally, flux-based and isotope-labelled metabolomics offers deeper insight into pathway dynamics rather than static metabolite levels, allowing identification of rate-limiting steps in key metabolic networks (Rao and Liu, 2025). However, metabolomics also has inherent limitations that can affect its application in crop improvement. The metabolome is highly sensitive to environmental fluctuations, developmental stages and sampling conditions, which can lead to variability and challenges in reproducibility (Razzaq et al., 2022). Comprehensive coverage of all metabolites remain difficult due to their vast chemical diversity, differences in polarity and concentration ranges of metabolites, often requiring multiple analytical platforms. Metabolite identification and annotation are also constrained by incomplete reference databases, leading to a significant proportion of unknown or poorly characterized compounds (Bharat et al., 2025). Additionally, sample preparation, instrument sensitivity and data processing pipelines can introduce technical biases, while the high cost and need for specialized expertise limit routine deployment in breeding programs (Agbona et al., 2022). In contrast, phenomics provides significant advantages by enabling high-throughput, non-destructive and precise measurement of plant traits across spatial and temporal scales. Phenomics enables the capture of complex traits, including plant architecture, biomass accumulation, photosynthetic efficiency and stress adaptation, which are difficult to assess through conventional methods (Jadhav et al., 2024). Importantly, it strengthens the genotype-phenotype linkage by generating large scale, quantitative trait datasets that can be directly integrated with genomic and other omics data. This integration enhances the accuracy of selection, supports genomic prediction models and accelerates the development of climate-resilient crop varieties through data-driven breeding strategies (Thingujam et al., 2025).

### 4.4 Role of phenomics in understanding millet growth and adaptation

Phenomics provides a powerful platform for dissecting such traits by integrating imaging technologies, automated sensors and data analytics (Mansoor et al., 2025). Phenotyping involves the use of standardized methods to measure morphological, physiological, anatomical and canopy level traits at the plant or population scale. Traditional phenotyping in breeding relies on visual assessment, touch and taste, making it time-consuming, labor-intensive and often destructive, particularly for large populations (Bian et al., 2022; Jadhav et al., 2024). In contrast, modern HTP employs fully automated, non-destructive sensor based systems capable of screening thousands of plants or field plots across hundreds of genotypes, with data automatically processed and archived for downstream analysis (Angidi et al., 2025). Additionally, various HTP systems have been developed for both controlled and field environments, employing imaging modalities such as visible (RGB) imaging, thermal infrared imaging, fluorescence, hyperspectral imaging and Light Detection and Ranging (LiDAR) (Zhou et al., 2022). These tools enable the precise measurement of plant growth, water use efficiency, canopy temperature, chlorophyll content, leaf architecture and photosynthetic performance. In foxtail millet, which serves as a model species for C4 grasses due to its small genome and short life cycle, phenomic platforms have been used to study dynamic responses to drought and nutrient stress (Wen et al., 2023). Under abiotic stress conditions, phenomic studies have elucidated the physiological traits that contribute to tolerance. In drought-tolerant finger millet genotypes, phenotyping revealed stable leaf area index (LAI), higher relative water content and reduced canopy temperature compared to susceptible varieties (Panda et al., 2023). Similarly, salinity phenotyping in foxtail millet demonstrated that tolerant lines maintain higher chlorophyll fluorescence (Fv/Fm ratio) and sustained photosynthetic performance under salt stress (Rashid et al., 2023). HTP has also been used to assess responses to heat stress, where parameters such as canopy temperature, chlorophyll index and leaf spectral reflectance provide early indicators of thermotolerance in finger millet (Angidi et al., 2025).

The advancements in phenomics are transforming traditional phenotyping into a high-resolution, predictive and decision support system that directly accelerates breeding progress. One of the most innovative ideas is field based high-throughput phenomics, where UAVs (drones), ground-based sensors and imaging platforms capture dynamic traits related to growth, canopy architecture, photosynthetic efficiency and stress responses under real field conditions, thereby reducing the gap between controlled environments and farmers’ fields (Oberson et al., 2024; Angidi et al., 2025). However, phenomics also faces several limitations that may constrain its large-scale implementation. HTP platforms require substantial initial investment, sophisticated infrastructure and skilled personnel for operation and maintenance. Data acquisition often generates massive, high-dimensional datasets that demand advanced computational tools, storage capacity and expertise in data analytics. Standardization across platforms, environments and protocols remains a major challenge, affecting data comparability and reproducibility (Yuan et al., 2023). Moreover, environmental and field heterogeneity can complicate trait interpretation and certain complex traits may still be difficult to capture accurately using current sensor technologies. In contrast, genetic engineering offers significant advantages by enabling precise and targeted manipulation of genes associated with desirable traits. Genetic engineering facilitates the rapid development of crop varieties with enhanced stress tolerance, improved nutritional quality, and resistance to pests and diseases (KhokharVoytas et al., 2023). Furthermore, the ability to engineer regulatory elements and pathways provides opportunities to fine-tune gene expression and optimize complex traits, making genetic engineering a powerful tool for next-generation, climate-resilient crop improvement strategies.

## 5 Genetic engineering approaches for trait enhancement in millets

**Figure 4:**
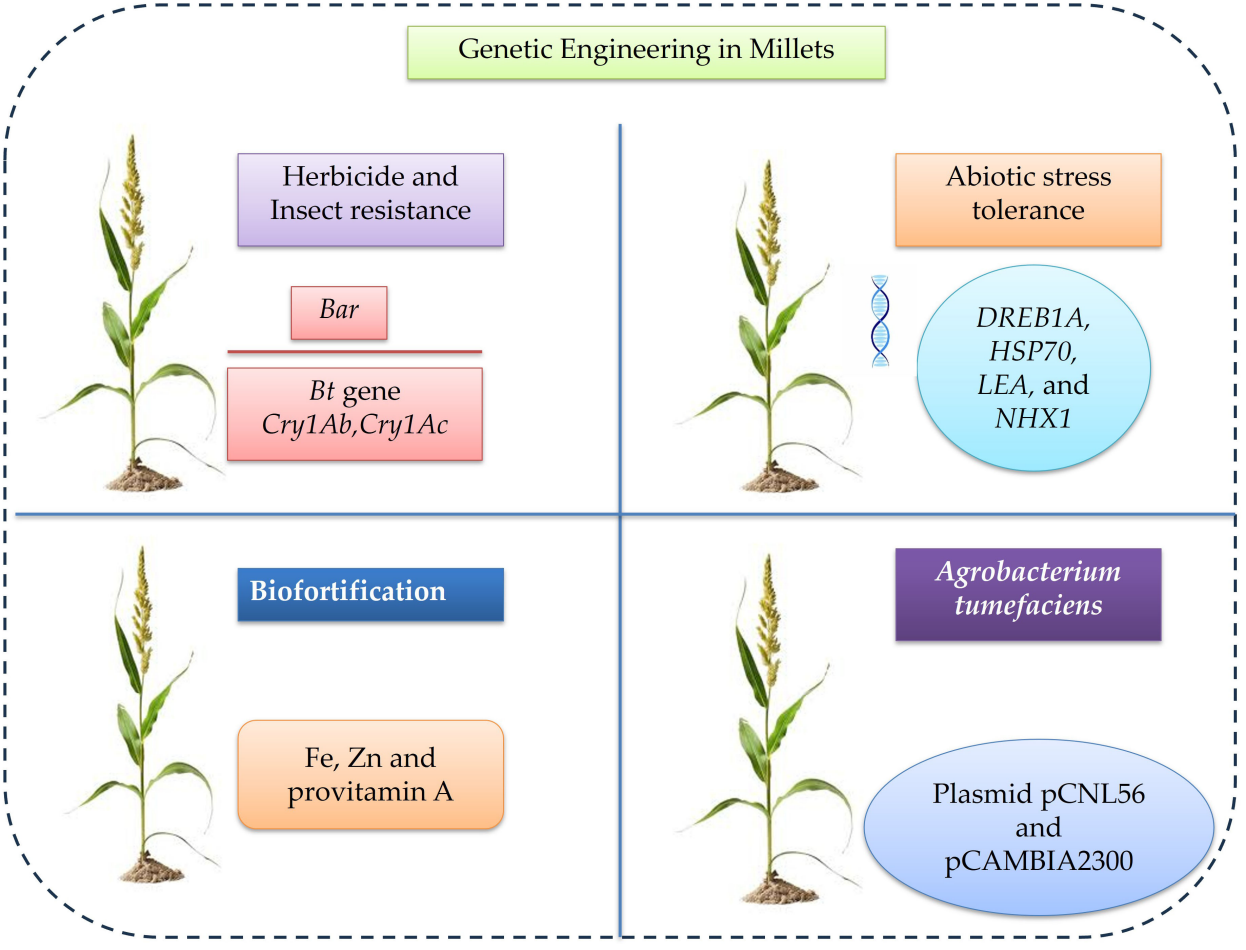
Schematic representation illustrates genetic engineering approaches for the identification and functional characterization of stress-responsive mechanisms in millets.

Genetic engineering has emerged as a transformative approach in millet improvement, enabling precise manipulation of the genome to introduce or modify desirable traits that are difficult to achieve through conventional breeding (Weldemichael and Gebremedhn, 2025). Millet transformation has been successfully achieved using both Agrobacterium tumefaciens-mediated and biolistic (gene gun) methods, which enable stable gene integration and expression (Krishna et al., 2025). Transgenic pearl millet plants expressing the bar gene for herbicide resistance and *Bacillus thuringiensis* (*Bt*) genes (*Cry1Ab*, *Cry1Ac*) (**Figure 4**) for insect resistance have been developed, demonstrating the feasibility of genetic transformation in these crops (Majumder et al., 2025). Similarly, the transformation of finger millet with marker genes such as *hpt* and *gusA* has proven stable gene transfer and expression systems in millets (Sapara et al., 2024). Abiotic stress tolerance is one of the significant targets of millet genetic engineering because these crops often face drought, salinity, and temperature extremes (Joshi, 2023). The introduction of stress-responsive genes such as *DREB1A, HSP70, LEA* and *NHX1* have been shown to improve tolerance to drought and salinity. For instance, a transgenic finger millet expressing the *DREB1A* gene under a stress-inducible promoter exhibited enhanced tolerance to drought, improved growth and higher yield compared to non-transgenic plants (Nair and Devi, 2024; Das et al., 2022). Although millets are naturally rich in calcium, iron, zinc and essential amino acids, genetic modification can further enhance their nutrient content and bioavailability (Kumar et al., 2022). Biofortification through transgenic approaches aims to increase levels of micronutrients, such as iron, zinc and provitamin A (**Figure 4**).

The overexpression of ferritin and nicotianamine synthase genes has been reported to enhance iron storage and transport in millet grains, while the expression of *phyA* gene has helped reduce phytic acid, which is an antinutritional factor limiting mineral absorption (Padmavathi et al., 2024). An efficient and reproducible *Agrobacterium tumefaciens* mediated genetic transformation protocol was established for kodo millet (*Paspalum scrobiculatum* L.) through optimization of key parameters. Among the tested strains, EHA105 and LBA4404 carrying plasmids pCNL56 and pCAMBIA2300, respectively, exhibited the highest transformation efficiencies. The inclusion of acetosyringone in the infection medium (200 µM for EHA105 and 250 µM for LBA4404) and in the co-cultivation medium (50 µM) further enhanced transformation efficiency (Bhatt et al., 2021). These genetic modifications can contribute to addressing malnutrition and micronutrient deficiencies among populations dependent on millet-based diets. However, genetic engineering also faces several important limitations. Regulatory approval processes are often lengthy, complex and vary significantly across countries, which can delay the deployment of engineered crops. Public perception and acceptance remain major challenges, particularly in regions where concerns about biosafety, environmental risks and ethical considerations persist (Yadav et al., 2024b). There is also the potential for unintended effects, such as off-target gene interactions or pleiotropic impacts on plant metabolism, which requires rigorous validation. Furthermore, high development costs, intellectual property issues and limited accessibility for small scale breeding programs can restrict equitable adoption. In contrast, genome editing offers distinct advantages by enabling more precise, efficient and often transgene free modifications (Akinbo et al., 2025). Tools such as CRISPR/Cas9 allow targeted alterations in native genes without introducing foreign DNA, which may simplify regulatory pathways and improve public acceptance in some contexts. A simple illustrative example can help clarify its potential, imagine a millet breeder working in a drought-prone region who identifies a native gene associated with water use efficiency through multi-omics analysis (Tyumentseva et al., 2023). Instead of introducing an external gene, genome editing is used to fine-tune this existing gene, slightly enhancing its expression so that the plant conserves water more effectively during stress. The resulting variety maintains yield under limited rainfall without altering its fundamental genetic identity. Farmers adopt this improved millet because it performs reliably under erratic climate conditions and consumers accept it more readily since no foreign genes were introduced (Kheya et al., 2023). Such precision, speed and flexibility make genome editing a powerful bridge between fundamental discovery and practical breeding, particularly for improving nutritional quality, stress resilience and sustainability in millet-based production systems.

## 6 Genome editing approaches for trait improvement in millets

**Figure 5:**
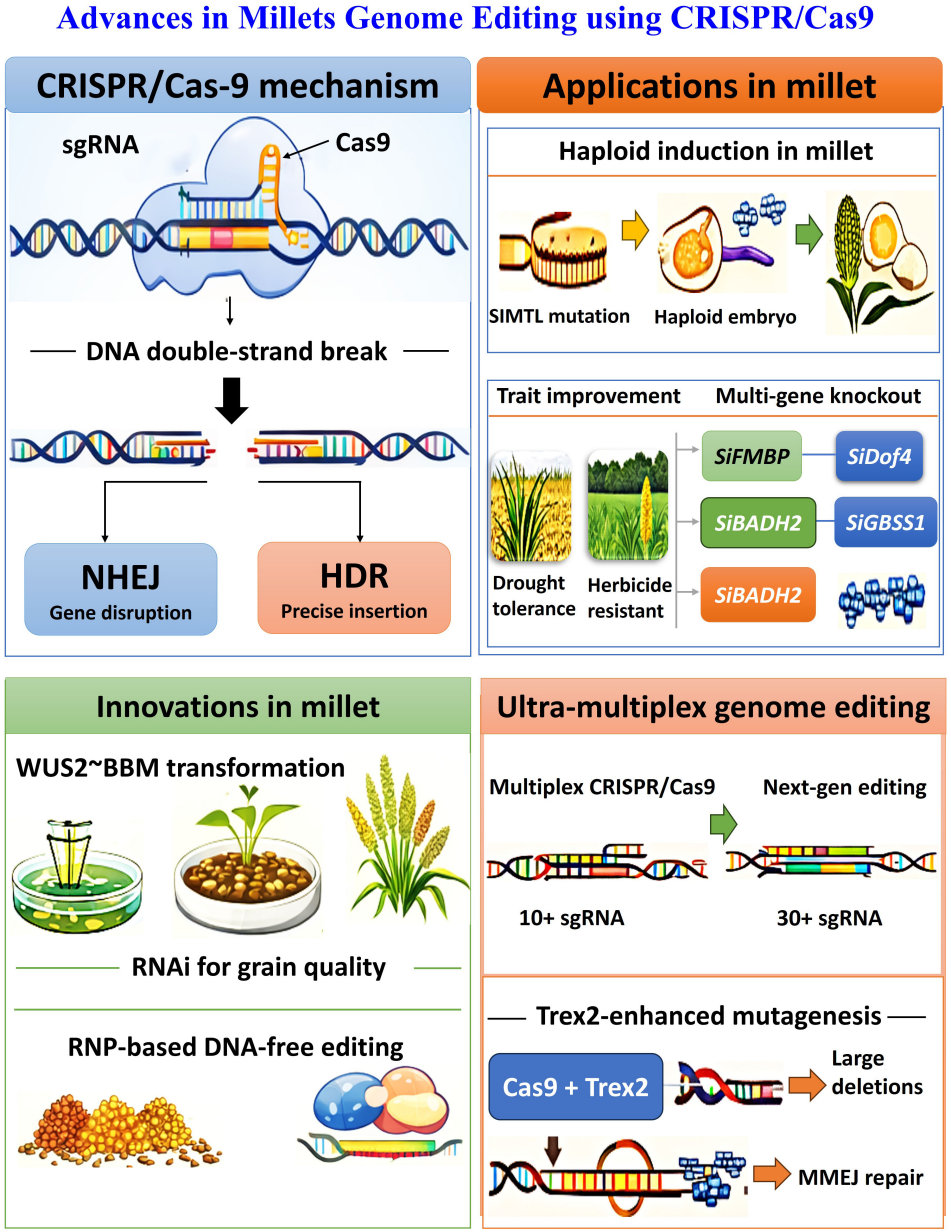
Schematic illustration depicting genome editing approaches employed to enhance stress adaptation and nutritional traits in millets.

Genome editing techniques offer a precise and efficient approach to identify, edit and transfer genes in millets which enhance its production and productivity. There are several techniques of genome editing such as CRISPR, mega-nuclease, base editing (BE), prime editing (PE), transcription activator-like endonuclease (TALENs) and zinc-finger nuclease (ZFNs), have been used targeting different traits (**Figure 5**) (Bhagtaney and Sundarrajan, 2023; Kumar et al., 2024a). These cutting edge biotechnology tools show great promise in improving growth and development as well as increasing resilience to stress (Hu et al., 2024). Among these techniques, the RNA-guided CRISPR/Cas9 system is an effective and versatile tool for heritable genome modification (Dutta et al., 2024), enabling the targeted modification of specific genes by introducing double-stranded breaks at desired genomic locations (Li et al., 2024). The Cas9 endonuclease, directed by a synthetic single-guide RNA (sgRNA), recognizes and cleaves target DNA sequences located adjacent to a protospacer adjacent motif (PAM) (Ma et al., 2015; Gupta et al., 2021). The resulting DNA break is repaired by either non-homologous end joining (NHEJ) or homology-directed repair (HDR), leading to gene disruption or precise insertion of desired genetic sequences (Ruis et al., 2025).

**Figure 6:**
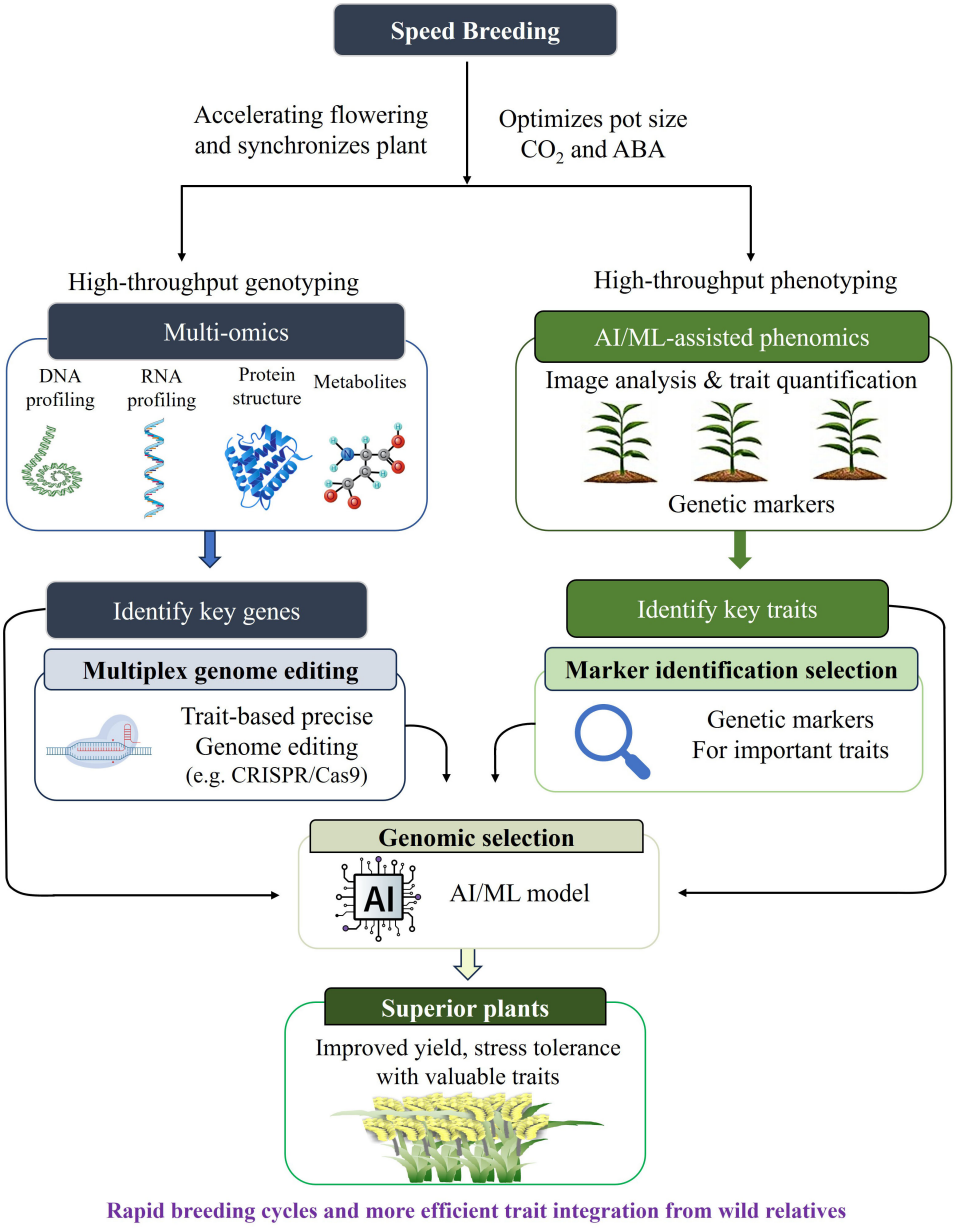
Schematic representation of integration of omics technology with speed breeding and machine learning.

In millets, CRISPR/Cas based genome editing has been increasingly explored for enhancing traits and analyzing functional genes (Panda et al., 2024). In a study, the potential off-target sites in pearl millet and finger millet were analyzed using BLAST searches with the 20 bp gRNA and adjacent 3 bp PAM sequences. For each gRNA, three putative off-target sites were identified, and six primer sets were designed to amplify regions flanking these sites. Approximately 520–570 bp genomic fragments surrounding each potential off-target region were amplified from T_1_ plants. PCR was conducted for both the edited plants and control plants transformed with the pCAMBIA-GRF: GIF: Cas9 construct, which lacks gRNAs, to assess off-target effects (Moin et al., 2025). Foxtail millet, a model species for C4 photosynthesis and stress biology, has been one of the first millets to benefit from CRISPR technology (Cheng et al., 2021). They reported the successful induction of haploid embryos from seeds through CRISPR/Cas9 mediated mutation of the *SiMTL* gene in foxtail millet. The *SiMTL* gene is an ortholog of the maize *MATRILINEAL/NOT LIKE-DAD/PHOSPHOLIPASE A* (*MTL/NLD/ZmPLA*) gene, which is known to play a crucial role in haploid induction (Kelliher et al., 2017; Yao et al., 2025; Sheng et al., 2025). In a study, Liang et al., 2022 employed the CRISPR/Cas9 single and multi gene knockout system to target five genes, *SiFMBP*, *SiDof4*, *SiBADH2*, *SiGBSS1* and *SiIPK1* in foxtail millet protoplasts to identify highly efficient sgRNAs. Homozygous mutant plants for most target genes were subsequently generated via Agrobacterium mediated transformation (Liang et al., 2022). The T_0_ generation exhibited a mutagenesis frequency of up to 100%, which was stably inherited by the next generation. No off-target mutations were detected at predicted sites. Furthermore, successful base editing was achieved using cytosine base editor and adenine base editor systems targeting *SiALS* and *SiACC* genes (Liang et al., 2022).

Notably, CBE mediated editing of *SiALS* resulted in a homozygous herbicide-tolerant mutant plant (Liang et al., 2022). By integrating existing RNA-seq data with functional information from model plants and other cereals such as maize and barley, candidate *ERFVII* genes were identified from the foxtail millet genome. This approach provides valuable targets for engineering waterlogging tolerance in foxtail millet through molecular and genetic interventions (Abdullah et al., 2021). The successful editing of genes associated with plant architecture, such as *SiGW2* and *SiBRI1*, stress tolerance and key metabolic pathways has confirmed the feasibility and efficiency of precise genome editing in foxtail millet (Panchal et al., 2023). These studies highlight the potential of CRISPR/Cas in uncovering gene functions and accelerating breeding programs (Mmbando, 2025). In sorghum, key innovations include the discovery of agronomic trait genes *WUS2–BBM* mediated genotype-independent transformation, RNAi for grain quality enhancement, CRISPR/transgene free genome editing and emerging nanobiotechnological tools for precise crop improvement (Wang et al., 2026a). Furthermore, haploid induction mediated genome editing and DNA-free editing using ribonucleoprotein (RNP) complexes accelerate the development of transgene-free varieties, thereby improving regulatory acceptance (Namo, 2023). Additionally, a key emerging concept is multiplex and pathway-level genome editing, in which multiple genes or regulatory elements within a biological pathway are edited simultaneously to fine tune complex traits such as drought tolerance, nitrogen use efficiency and disease resistance (Wang et al., 2025b). Ultra-multiplex CRISPR platforms represent a major advance in plant genome engineering, enabling the simultaneous targeting of dozens of loci, with scalability primarily constrained by vector capacity and sgRNA expression efficiency (**Table 2**). Conventional multiplex systems commonly accommodate more than 10 sgRNAs, whereas next-generation platforms have greatly expanded this limit (Bindal and Rath, 2025). Therefore, ultra-multiplex editing strategies provide an unprecedented framework for combinatorial trait engineering, facilitating one step optimization of yield architecture, metabolic flux and stress-responsive pathways while accelerating large-scale genotype-to-phenotype discovery (Kumar et al., 2025).

**Table 2:** Current status of genome editing in millets: technological advances, trait targets and challenges.

Genome editing tool	Millet species	Target gene	Trait	Reference
Off-target validation of CRISPR	Pearl millet and Finger millet	gRNA-specific predicted loci	Editing specificity assessment	Moin et al., 2025
CRISPR/Cas9-mediated haploid induction	Foxtail millet	*SiMTL*	Haploid embryo induction	Cheng et al., 2021
Single and multiplex CRISPR knockout	Foxtail millet	*SiFMBP, SiDof4, SiBADH2, SiGBSS1 and SiIPK1*	Functional genomics and trait discovery	Liang et al., 2022
Cytosine and adenine base editing	Foxtail millet	*SiALS* and *SiACC*	Herbicide tolerance and precise nucleotide conversion	Liang et al., 2022
CRISPR	Foxtail millet	*SiGW2, SiBRI1* and metabolic genes	Plant architecture, stress tolerance and pathway engineering	Panchal et al., 2023
Candidate-gene discovery for editing	Foxtail millet	ERFVII family genes	Waterlogging tolerance	Abdullah et al., 2021
Genotype-independent transformation-linked editing	Sorghum	WUS2-BBM assisted platforms	Agronomic trait improvement	Wang et al., 2026a
Multiplex and pathway-level editing	Millets	Multiple pathway genes	Drought tolerance, NUE and disease resistance	Wang et al., 2025b
Ultra-multiplex CRISPR platforms	Millets	Dozens of loci	Combinatorial trait engineering	Bindal and Rath, 2025; Kumar et al., 2025
Cas9-Trex2 mediated multiplex editing	Green foxtail	Multiple loci	Large deletion engineering and editing efficiency	Weiss et al., 2020; Tsai et al., 2020
Pangenome-assisted editing target discovery	Millets	Core and dispensable genes	Drought resilience	Yan et al., 2023; MacNish et al., 2026
Emerging alternatives	Millets	RNP-based systems	Overcoming regeneration bottlenecks	Shashi and Bhat, 2025; Li et al., 2026

For example, Weiss et al., 2020 developed a rapid protoplast assay to optimize multiplexed CRISPR/Cas9 editing in *S. viridis*. Co-expression of the exonuclease Trex2 enhanced targeted mutagenesis efficiency by an average of 1.4-fold. The Cas9-Trex2 system generated a distinct mutation spectrum, with 94% of deletions exceeding 10 bp and virtually no insertions across tested targets. Further analysis showed that 52.2% of Cas9-Trex2-induced deletions were repaired via microhomology-mediated end joining (MMEJ), compared with only 3.5% for Cas9 alone, indicating a strong shift from canonical non-homologous end joining (Weiss et al., 2020). Trex2-mediated exonucleolytic resection exposes microhomologous sequences around Cas9-induced DSBs, thereby suppressing classical NHEJ and promoting MMEJ-driven larger deletions with minimal insertions (Tsai et al., 2020).

However, editing efficiency can vary depending on the target gene, species and delivery system and many crop plants, particularly minor millets still face challenges related to transformation and regeneration. In addition, incomplete knowledge of gene functions and complex gene interactions may lead to unintended phenotypic consequences. Regulatory uncertainty in some regions, along with technical costs and infrastructure requirements, can further limit widespread adoption, especially in resource-constrained breeding programs (Sapara et al., 2024). A critical limitation to the practical deployment of genome editing technologies in millets is the challenge of efficient transformation and plant regeneration systems. Unlike major cereals such as rice and maize, many millet species exhibit a high degree of recalcitrance to *in vitro* culture, which significantly constrains the successful integration and expression of editing constructs (Ranjan et al., 2026; Pandey et al., 2026). This recalcitrance is often compounded by a strong genotype dependency, where only a limited number of cultivars respond favorably to tissue culture conditions, thereby restricting the broader applicability of optimized protocols (Singh et al., 2026). Moreover, existing transformation methods, including *Agrobacterium* mediated and biolistic approaches, often exhibit low efficiency and poor reproducibility across different laboratories and species (Kumar et al., 2021; Sharma et al., 2022). Since genome editing platforms such as CRISPR/Cas systems fundamentally rely on the efficient delivery of editing reagents into plant cells, followed by regeneration into whole plants, these limitations directly impede the scalability and translational potential of such technologies in millet improvement programs. Additionally, prolonged tissue culture phases often introduce somaclonal variations, further complicating trait stabilization and downstream breeding efforts. Despite recent advances, such as the use of morphogenic regulators, improved explant selection and optimization of culture media, a robust, genotype independent transformation system remains elusive for most millets (Bhat et al., 2023). Emerging alternatives, including in planta transformation and DNA-free editing via ribonucleoprotein complexes, offer promising avenues to circumvent traditional bottlenecks; however, their applicability in millets is still at an early stage and requires further validation (Li et al., 2026). Therefore, addressing these foundational constraints is essential to bridge the gap between theoretical advances in genome editing and their practical application in millet breeding, ensuring that these technologies can be effectively harnessed for crop improvement under diverse agro-ecological conditions.

However, the advancements in genome editing are moving beyond single gene knockouts toward precision, multiplexed and climate responsive genetic redesign. One of the most significant advances are next-generation CRISPR technologies, such as prime editing, which enable precise nucleotide substitutions or small insertions without creating double strand breaks, allowing accurate modification of elite alleles associated with yield, quality and stress tolerance (Saber Sichani et al., 2023). Editing of cis-regulatory elements, promoters, enhancers and untranslated regions (UTRs) represents a novel strategy to modulate gene expression quantitatively rather than switching genes on or off, thereby mimicking natural variation (Cui et al., 2023). In contrast, pangenomics offers significant advantages by capturing the full spectrum of genetic diversity within a species, including core and dispensable genes that are often missed in single reference genomes. This comprehensive view enables the identification of novel alleles, structural variations and presence or absence variations associated with important agronomic traits (MacNish et al., 2026). A simple illustrative example highlights its value: consider a breeder working on millet improvement that relies on a single reference genome and fails to detect a drought resilience gene absent from that reference but present in traditional landraces. Through pangenome analysis, this hidden gene is discovered across diverse farmer maintained varieties. The breeder then incorporates this allele into elite lines using modern breeding or genome editing approaches (Yan et al., 2023; MacNish et al., 2026). As a result, new millet varieties exhibit improved drought tolerance while maintaining yield stability. Farmers benefit from more resilient crops, and the breeding program gains access to previously untapped genetic resources. Thus, by revealing hidden genetic variation and enabling more informed trait discovery, pangenomics strengthens the foundation for precision breeding and enhances the effectiveness of downstream approaches such as GS and genome editing (Chhajer et al., 2026; Jacob et al., 2026).

## 7 Pangenome approaches for understanding genetic diversity in millets

The concept of pangenome has revolutionized modern plant genomics by providing a comprehensive representation of a species’ entire genetic repertoire, encompassing both core and variable genes across diverse genotypes (Hou and Pang, 2024). The development of pangenomes has opened new avenues for understanding genomic diversity, evolutionary dynamics and the genetic basis of key agronomic traits (Naithani et al., 2023). In the case of millets, the development of pangenomes is particularly valuable due to their wide genetic diversity, domestication histories and adaptation to harsh environments. The construction of pangenomes relies heavily on advances in high-throughput sequencing technologies, assembly algorithms and comparative genomics tools (Sarawad et al., 2025). Several millet species have undergone whole genome sequencing in the past decade, paving the way for pangenomic analyses (Maharajan et al., 2024). Among these, foxtail millet and pearl millet have received the most attention due to their well-assembled reference genomes and economic importance (Kumar et al., 2021). For foxtail millet, multiple genome assemblies from diverse accessions have been integrated to build a high quality pangenome, revealing substantial structural variations, including presence or absence variations, copy number variations and inversions (Wang et al., 2021). These variations contribute to phenotypic diversity in traits such as flowering time, grain yield and tolerance to abiotic stress (Gonzalez et al., 2022).

Similarly, pearl millet, known for its exceptional drought and heat tolerance, has been a focus of recent pangenomic studies (Yan et al., 2023). The inclusion of landraces and wild relatives in pangenome analyses have uncovered candidate genes associated with stress adaptation, nutrient use efficiency, and grain quality (Wang et al., 2026b). Structural variations identified through comparative genomics have revealed novel gene clusters related to disease resistance, hormone signaling and metabolic pathways that were absent in the reference genome (Zhou et al., 2023). For finger millet, which possesses an allotetraploid genome, pangenome studies are still emerging due to its complex genome architecture (Liang and Han, 2024). However, initial efforts combining transcriptome data and resequencing of diverse accessions have provided insights into gene family expansion and adaptive evolution. Similar efforts in proso millet and barnyard millet are uncovering species-specific genes associated with early maturation, salinity tolerance and nutritional enhancement (Tay Fernandez et al., 2022). In a study, a comprehensive pan-genomic analysis of stress-associated protein (SAP) genes was performed in pearl millet, through a combination of keyword searches, Hidden Markov Model (HMM) profiling and BLASTP analysis, 16–23 PgSAP genes were identified across 11 pearl millet genomes and classified into 22 orthologous gene groups (OGGs). Phylogenetic analysis categorized these genes into nine subfamilies, with core OGGs present in all genomes exhibiting greater evolutionary conservation than dispensable OGGs, as evidenced by lower Ka/Ks ratios (Zhang et al., 2025). In a study, the phytochrome A signal transduction 1 (PAT1) subfamily of GRAS transcription factors play crucial roles in regulating responses to various abiotic stresses; however, their involvement in light-mediated temperature stress remains poorly understood. In this study, 106 PAT1-GRAS genes were identified across 11 pearl millet accessions. Gene structure (exon-intron organization) and conserved protein motifs were highly preserved among the PAT1-GRAS members. Variation in gene duplication events and chromosomal localization suggested evolutionary divergence of PAT1-GRAS genes among the accessions. Cis-regulatory element analysis suggested potential roles of PAT1-GRAS genes in responses to light and temperature stress. Key PAT1-GRAS members were further characterized for their involvement in light-mediated temperature stress regulation (Xie et al., 2024). The SRS gene family was systematically investigated in foxtail millet using pan-genome data from 110 core germplasm resources along with two high-quality reference genomes (XM and Yu1). SRS family members were identified and their intra-species distribution patterns, including copy number variations and inter-chromosomal translocations, were analyzed. A novel standardized nomenclature system was proposed to unify gene family naming, facilitating direct visualization of gene copy number variation across germplasms and enabling the identification of core and variable members while highlighting chromosomal translocation events (Li et al., 2025).

The advancements in pangenomics are redefining how genetic diversity is discovered, interpreted and utilized by moving beyond single reference genomes toward diversity aware, function-driven breeding strategies. A key innovative idea is the construction of graph-based pangenomes, which capture structural variants, presence or absence variations, copy number variations and novel genes from elite cultivars, landraces and wild relatives, enabling more accurate genotype-phenotype associations for complex traits (Hameed et al., 2022). An emerging concept is functional pangenomics, where core and dispensable genes are linked with transcriptomic, proteomic and metabolomic data to identify condition specific and stress-inducible genes that are often absent from reference genomes but critical for adaptation and resilience (Abdullah et al., 2022). This is particularly valuable for traits such as disease resistance, abiotic stress tolerance and nutrient efficiency. Another novel strategy is trait and environment-specific pangenomes, developed for specific agro-climatic zones or stress conditions, allowing breeders to identify adaptive alleles relevant to local environments.

## 8 Speed breeding in millet

Speed breeding is an advanced crop improvement strategy that accelerates the breeding cycle and facilitates the rapid development of improved varieties (Hickey et al., 2017). It involves the deliberate manipulation of environmental factors such as light intensity, photoperiod, temperature and humidity to promote faster plant growth and an earlier transition from vegetative to reproductive stages. Unlike conventional breeding approaches, which typically require several cropping seasons to achieve homozygosity and stabilize desirable traits, speed breeding significantly shortens generation time (Ferrante and Mariani, 2018). This technique commonly employs extended photoperiods of 20-22 hours of light per day, along with optimal temperature regimes of 25 to 30 °C and controlled humidity. These optimized environments enhance photosynthetic activity, stimulate rapid vegetative development and induce early flowering, thereby reducing the time required to complete each generation (Kolad et al., 2025). In millets, the application of speed breeding enables the completion of approximately 4 to 6 generations per year, compared to only 1 to 2 generations under conventional field conditions. This technique is particularly valuable in millet improvement programs targeting climate resilience, nutritional enhancement and yield stability under stress-prone environments (Sharma et al., 2024). For instance, traits such as drought tolerance, heat resilience and biofortification can be rapidly introgressed and fixed using speed breeding in combination with MAS or GS. For example, this approach holds significant potential for developing durable and long lasting resistance against the blast disease pathogen in finger millet (Mbinda and Masaki, 2021). They developed a cost effective, repeatable speed breeding protocol for finger millet by optimizing agronomic and physiological conditions, thereby reducing crop duration by 28–54 days. The protocol enabled 4–5 generations per year, compared to 1–2 under field conditions and was successfully validated in segregating populations (Sajja et al., 2025). They optimized a speed breeding protocol for pearl millet and wild Pennisetum species by refining pot size, CO_2_ enrichment and exogenous ABA application, thereby accelerating flowering, synchronizing plant development and reducing generation time. This improved protocol enables rapid cycling of breeding populations and faster introgression of valuable traits from wild relatives (Terada et al., 2026). Efforts to accelerate generation advancement through speed breeding and rapid generation advancement (RGA) techniques have already been explored in minor millets and related cereals to shorten breeding cycles. For example, Rizal et al., 2014 employed a split-culm approach combined with embryo rescue in sorghum, successfully reducing the generation time to 88 days compared with the conventional 120 days (Rizal et al., 2014). Moreover, the integration of MAS with speed breeding has further transformed breeding pipelines by enabling early-generation selection under controlled rapid growth conditions, drastically reducing the time required to develop elite cultivars (Qiao et al., 2026).

## 9 Machine learning integration with multi-omics approaches in millet breeding

ML has emerged as a transformative tool in modern crop improvement, offering unprecedented opportunities to integrate multi-omics data with conventional breeding strategies (Syeda, 2025). ML is increasingly being applied to enhance breeding efficiency, particularly when combined with MAS, transcriptomics, proteomics, phenomics, metabolomics, genetic engineering, CRISPR-based genome editing and speed breeding. These integrative approaches facilitate a deeper understanding of complex traits such as drought tolerance, heat resilience, nutrient use efficiency and grain quality, which are critical for millet improvement under changing climatic conditions (Jadhav et al., 2024; Das et al., 2026). In this context, MAS has long served as a cornerstone of molecular breeding by utilizing genetic markers linked to desirable traits. However, its application in millets has often been constrained by limited genomic resources and the polygenic nature of key agronomic traits (**Figure 6**) (Ingle et al., 2023). ML addresses these limitations by capturing complex, non-linear relationships between genotypic and phenotypic data. Algorithms such as support vector machines and gradient boosting can efficiently process high dimensional SNP datasets, enabling more accurate prediction of complex trait performance compared to traditional statistical approaches (Jakhmola-Mani et al., 2024). Building on this capability, ML-driven GS selection models are increasingly being applied in millets to predict breeding values at early stages of plant development. This early and precise prediction not only accelerates selection decisions but also reduces the time and cost associated with conventional breeding cycles, thereby enhancing the overall efficiency of millet improvement programs (Sinha et al., 2023; Kumar and Walia, 2024). Furthermore, integrating ML with MAS improves marker discovery and validation by identifying interactions among multiple loci, including epistatic effects that are often overlooked in conventional QTL mapping or GWAS. In addition, ML facilitates the identification of candidate genes for marker development and functional validation. Supervised learning models can classify genotypes based on stress response profiles, enabling the selection of superior breeding lines. Feature selection techniques integrated within ML pipelines help identify the most informative markers, thereby reducing redundancy and improving model efficiency (Kassem, 2025; Essemine et al., 2026). This is particularly important in millets, where complex LD patterns and population structure can confound association analyses.

ML also plays a critical role in transcriptomic data analysis. Large-scale gene expression datasets can be effectively analyzed using clustering algorithms and dimensionality reduction techniques such as PCA and t-distributed stochastic neighbor embedding (t-SNE) (Dutta et al., 2026). Advanced ML models, including deep learning architectures, further enable the reconstruction of gene regulatory networks and the identification of key transcription factors involved in stress-responsive pathways, thereby providing a mechanistic understanding of how plants respond to environmental stresses (Dutta and Dutta, 2025). Complementing these insights, proteomics, which offers a direct view of functional protein dynamics, and also benefit significantly from ML based analysis, where complex protein expression patterns, interactions, and post-translational modifications can be efficiently decoded to uncover critical regulators of stress tolerance and adaptation (Kumar et al., 2025). Proteomic datasets are inherently high-dimensional and often noisy, posing analytical challenges. ML techniques such as support vector machines and artificial neural networks facilitate protein classification, biomarker discovery and identification of differentially abundant proteins associated with stress tolerance and metabolic adaptation (Zhang et al., 2024).

Phenomics represents another domain where ML has had a substantial impact. Traditional phenotyping approaches are labor-intensive and often subjective, limiting their scalability. In contrast, ML powered phenomics leverages high-throughput imaging technologies, remote sensing and sensor-based platforms to capture detailed phenotypic traits across spatial and temporal scales (**Figure 6**). In millets, traits such as plant height, biomass, canopy temperature, leaf area and stress responses can be quantified using drones, hyperspectral imaging and thermal sensors, thereby enabling precise and objective phenotypic assessments (Angidi et al., 2025). Similarly, metabolomics has greatly benefited from ML applications. However, the complexity and variability of metabolomic data necessitate advanced analytical approaches. ML techniques, including unsupervised methods such as hierarchical clustering and PCA, as well as supervised classification models, are widely used to identify metabolite patterns and classify samples (Shawky et al., 2025; Yan and Wang, 2022). Additionally, automated imaging systems coupled with machine learning algorithms can capture temporal variations in growth rate, leaf angle and color indices to quantify stress effects and recovery patterns (Wen et al., 2023). Similarly, in pearl millet, thermal and hyperspectral imaging have been applied to assess canopy temperature depression, relative water content and photosynthetic efficiency under heat and water stress, providing key indicators for identifying drought-tolerant genotypes (Krishna et al., 2021; Almeida et al., 2022).

Network-based ML approaches further allow the identification of hub genes that play central roles in stress adaptation, providing valuable targets for genetic engineering and genome editing technologies such as CRISPR (Yan and Wang, 2023; Amin et al., 2025). Despite its significant potential, the application of ML in millet research faces several challenges. A major limitation is the scarcity of high-quality, large-scale datasets. Compared to major cereals such as rice, wheat and maize, millets have relatively underdeveloped genomic and phenotypic resources. Issues related to data standardization, integration and sharing further hinder ML model performance (Ghatak et al., 2025). Additionally, the interpretability of complex ML models remains a concern, particularly in biological systems where mechanistic understanding is essential. Addressing these challenges requires coordinated efforts to generate comprehensive, high quality multi omics datasets for millets. Advances in next-generation sequencing, HTP and bioinformatics are expected to improve data availability and quality. Moreover, the development of explainable artificial intelligence (AI) approaches will enhance the transparency and interpretability of ML models, thereby increasing their utility in biological discovery and breeding decision-making (Banshidhar et al., 2023; Mane et al., 2024).

Therefore, machine learning is revolutionizing millet breeding by enabling the integration of marker-assisted selection with multi-omics technologies. By harnessing genomics, transcriptomics, proteomics, phenomics and metabolomics, ML provides a holistic framework for understanding and improving complex traits (Aggarwal et al., 2025: Amin et al., 2025). These advancements are particularly crucial for millets, which play a vital role in ensuring food and nutritional security under climate change. As data resources and computational methodologies continue to evolve, ML-driven multi-omics integration is expected to play a central role in the development of high-yielding, stress-resilient and nutritionally enhanced millet varieties (Yang et al., 2025; Zaidi et al., 2026).

## 10 Challenges and future perspective

Despite rapid advancements in genomic technologies and breeding tools, several critical challenges continue to limit their effective deployment in millet improvement programs. One of the foremost bottlenecks is the inefficiency of transformation and regeneration systems. Many millet species remain highly recalcitrant to *in vitro* culture, with strong genotype dependent responses and poor responsiveness to established protocols. This significantly constrains the application of genome editing technologies, such as CRISPR/Cas systems, which rely on efficient delivery of editing reagents and subsequent regeneration of whole plants. Furthermore, low transformation efficiency, lack of reproducibility across laboratories and the risk of somaclonal variation during prolonged tissue culture further complicates the development of stable edited lines. Although emerging approaches, including morphogenic regulator-assisted transformation, in planta methods and DNA-free editing using ribonucleoprotein complexes, offer promising alternatives, their scalability and consistency across diverse millet species remain to be fully established. In addition to technical constraints, the translational pipeline from gene discovery to cultivar development remains inadequately addressed. Functional validation of candidate genes is often limited, with insufficient emphasis on systematic pipelines that include gene editing, transgenic approaches, and loss and gain-of-function studies to confirm trait associations. Moreover, while multi-omics approaches including genomics, transcriptomics, proteomics and metabolomics are increasingly employed for trait dissection, their integration into coherent operational breeding pipelines is still in its infancy. Bridging this gap requires the development of robust frameworks that link omics-derived insights with MAS and GS strategies. The incorporation of AI and ML into breeding programs presents a transformative opportunity to enhance prediction accuracy and accelerate selection cycles. AI-driven platforms can integrate large-scale, multi-dimensional datasets to enable predictive breeding and decision support systems. However, their practical implementation in millets remains limited, often constrained by inadequate datasets, a lack of standardized phenotyping and gaps in computational infrastructure. Equally important is the need for rigorous field level validation of improved genotypes under diverse agro ecological conditions to ensure trait stability, adaptability and farmer acceptance. Beyond scientific and technical considerations, regulatory and biosafety frameworks play a decisive role in the deployment of genome edited crops. Variability in regulatory policies across countries, coupled with concerns regarding biosafety and public perception, can influence the adoption and dissemination of these technologies. Finally, the successful translation of research innovations into farmer ready solutions depends on well defined commercialization pathways. This includes varietal testing, seed system development, intellectual property management and strong public private partnerships to facilitate large scale dissemination. Moving forward, a holistic and integrated approach that addresses technical, biological, computational, regulatory and socio-economic dimensions will be essential to fully harness the potential of next-generation breeding technologies for sustainable millet improvement under changing climatic conditions.

## 11 Conclusion

The rapid advancement of modern technologies has revolutionized millet breeding, unlocking unprecedented opportunities to enhance productivity, nutritional quality and resilience to environmental stresses. Millets, once regarded as orphan crops, are now emerging as pivotal components of climate-smart and sustainable agriculture owing to their inherent tolerance to drought, heat and low-input conditions. The integration of multi-omics approaches, including genomics, transcriptomics, proteomics, metabolomics and epigenomics, has substantially deepened our understanding of the genetic architecture and molecular mechanisms governing complex traits across diverse millet species. Such integrative omics frameworks, when combined with advanced HTP platforms, enable precise dissection of genotype-phenotype relationships and facilitate the identification of candidate genes, regulatory networks and biomarkers associated with yield improvement, stress tolerance and nutritional enhancement.

In parallel, AI-driven analytical tools, machine learning algorithms and predictive breeding models are increasingly accelerating millet improvement by enabling rapid analysis of large scale omics and phenotypic datasets, improving trait prediction accuracy, and supporting data-driven decision-making in breeding pipelines. MAS, GWAS and GS have collectively enhanced genetic gains by enabling early, precise and large-scale selection of superior genotypes. Moreover, the development of high-quality reference genomes, pangenomes and haplotype resolved assemblies has broadened the accessible genetic diversity, allowing breeders to mine novel allelic variants and rare adaptive genes linked to productivity and environmental fitness. Furthermore, cutting-edge biotechnological interventions such as transgenic approaches and CRISPR/Cas-based genome editing, base editing and prime editing systems offer unprecedented precision for targeted trait manipulation, particularly for complex traits that are difficult to improve through conventional breeding alone. These tools have already shown remarkable promise in enhancing abiotic stress tolerance, nutrient use efficiency and pests and diseases resistance across multiple millet species. Looking ahead, future research should increasingly focus on the integration of pangenomics, GS, genome editing, advanced phenomics and AI-assisted breeding frameworks to develop next-generation millet cultivars. Such efforts will be crucial for designing climate-resilient breeding strategies capable of sustaining productivity under changing environmental conditions, thereby transforming millet improvement into a highly predictive, precision-based and resilient breeding paradigm.
