# Supplementary material for: Modern genomic and omics-based technologies for millet breeding and genetic improvement
Source: Front Plant Sci. 2026 May 20;17:1782766. doi: 10.3389/fpls.2026.1782766 (PMC13230146; doi:10.3389/fpls.2026.1782766)
Supplement: Supplementary file 1 [file Table1.docx]

**Supplementary Table 1. Molecular Marker-Assisted Identification of Disease-Resistant Traits in Millets: Summary of Marker Types, Populations, and Target Stresses.**

| **S.No.** | **Millet Name** | **Marker** | **Population** | **Stress Name** | **References** |
| --- | --- | --- | --- | --- | --- |
| 1 | Finger Millet | SNP | F_2_:3 | Blast | Pendergast et al., 2022 |
| 2 | Finger Millet | SNP | Diverse set | Blast | Sood et al., 2023 |
| 3 | Pearl Millet | SNP | Inbred | Blast | Singh et al., 2024 |
| 4 | Pearl Millet | SSR | Diverse | Blast | Bisoriya et al., 2025 |
| 5 | Pearl Millet | SNP | Advance breeding lines | Blast | Pujar et al., 2023 |
| 6 | Sorghum | Microsatellite | RIL | Shoot fly | Satish et al., 2009 |
| 7 | Pearl Millet | SSR | F_8_ RIL | Downy mildew | Chelpuri et al., 2019 |
| 8 | Pearl Millet | SNP | RIL | Nematode | Vutla et al., 2025 |
| 9 | Pearl Millet | AFLP and SSR | RIL | Nematode | Liu, 2012 |
| 10 | Pearl Millet | SSR | Germplasm lines | Blast | Rajpoot et al., 2023 |
| 11 | Pearl Millet | SSR | Germplasm | Downy mildew | Zala et al., 2017 |
| 12 | Pearl Millet | ISSR | Germplasm | Downy mildew | Parmar et al., 2022 |
| 13 | Pearl Millet | SCAR | F_2_ | Downy mildew | Jogaiah et al., 2014 |
| 14 | Pearl Millet | SNP | F6:7 RIL | Downy mildew | Liu et al., 2024 |
| 15 | Pearl Millet | EST-SSR | F7 RIL | Downy mildew | Rajaram et al., 2013 |
| 16 | Finger Millet | SSR | Germplasm | Blast Disease | Babu et al., 2014 |
| 17 | Finger Millet | SSR | Germpalsm | Blast Disease | Babu et al., 2018 |
| 18 | Finger Millet | SSR | Germpalsm | Blast Disease | Ojha et al., 2024 |
| 19 | Finger Millet | SNP | F_6_ RIL | Blast Disease | Tian et al., 2021 |
| 20 | Finger Millet | SSR | Germplasm | Blast Disease | Thakur et al., 2024 |
| 21 | Foxtail Millet | SNP | Germplasm | Blast Disease | Hui et al., 2021 |
| 22 | Sorghum | SNP | Germplasm | Ergot | Kebede et al., 2025 |
| 23 | Sorghum | SNP | Germplasm | Anthracnose | Birhanu et al., 2024 |
| 24 | Sorghum | SNP | Sorghum | Anthracnose | Ahn et al., 2025 |
| 25 | Sorghum | SSR | Germplasm | Striga hermonthica | Ahmed et al., 2025 |
| 26 | Sorghum | SSR | F_7_ RIL | foliar diseases | Murali et al., 2010 |
| 27 | Sorghum | SNP | Accession | Grain mold and rust | Upadhyaya et al., 2013 |
| 28 | Sorghum | microsatellites and AFLPs | F_5_ | Grain mold | Klein et al., 2001 |
| 29 | Sorghum | SNP | Germpalsm | Anthracnose | Mengistu et al., 2021 |
| 30 | Pearl millet | DArT + SSR | RIL | Drought & agronomic traits | Srivastava et al., 2020 |
| 31 | Foxtail millet | SSR + InDel | F₂ mapping | Drought | Qie et al., 2014 |
| 32 | Foxtail millet | SNP | RIL | Yield-related traits | Liu et al., 2020 |
| 33 | Proso millet | SNP | Diversity panel | Salinity | Boukail et al., 2021 |
| 34 | Little millet | EST-SSR | Transcriptome set | Drought tolerance | Desai et al., 2021 |
| 35 | Barnyard millet | GBS / SNP | Accessions | Abiotic resilience | Pradhan et al., 2024 |
| 36 | Pearl millet | DArT + SSR | Backcross lines | Terminal drought | Sehgal, 2016 |
| 37 | Little millet | SSR (cross-transfer) | Germplasm panels | Drought | Gautam et al., 2022 |
| 38 | Kodo millet | SSR / RAPD | Germplasm | Drought adaptation | Yadav et al., 2016 |
| 39 | Pearl millet | Gene-based SSRs | HRC (~2500) | Terminal drought | Sehgal et al., 2015 |
| 40 | Pearl millet | DArT + SSR | RIL | Rust / disease resistance | Ambawat et bal., 2016 |
| 41 | Finger millet | SNP/SSR high-density | Mapping population | Blast resistance | Gimode et al., 2016 |
| 42 | Foxtail millet | EST-SSR + InDel | Diverse germplasm | Various traits | Hariprasanna et al., 2025 |
| 43 | Proso millet | SSR | Germplasm diversity | Abiotic tolerance | Trivedi et al., 2015 |
| 44 | Kodo millet | RAPD / SSR | Germplasm | Drought adaptation | Goron and Raizada, 2015 |
| 45 | Barnyard millet | SSR / EST-SSR | Mapping population | Agronomic / stress traits | Babu and Chauhan, 2017 |
| 46 | Finger millet | SSR / SNP | Association panel | Nutritional trait (Ca) | Puranik et al., 2022 |
| 47 | Sorghum | SSR | Diverse germplasm panel | Drought | Motlhaodi et al., 2017 |
| 48 | Sorghum | SNP (GBS) | Biparental mapping population (RILs, 200 lines) | Heat | Engida, 2017 |
| 49 | Sorghum | RAD-seq SNP | Backcross-derived lines (BC populations) | Shoot fly (biotic) | Colasuonno et al., 2021 |
| 50 | Sorghum | KASP | Mutant / TILLING population | Nutrient deficiency (N) | Dampanaboina et al., 2019 |
| 51 | Sorghum | InDel | Core collection subset (100 accessions) | Waterlogging | Xin et al., 2021 |
